# Reprogramming the immunosuppressive tumor microenvironment results in successful clearance of tumors resistant to radiation therapy and anti-PD-1/PD-L1

**DOI:** 10.1080/2162402X.2023.2223094

**Published:** 2023-06-15

**Authors:** Debayan Mukherjee, Erminia Romano, Richard Walshaw, Leo A. H. Zeef, Antonia Banyard, Stephen J. Kitcatt, Eleanor J. Cheadle, Karoliina Tuomela, Swati Pendharkar, Aws Al-Deka, Beatrice Salerno, Sophie Raby, Ian G. Mills, Jamie Honeychurch, Tim M. Illidge

**Affiliations:** a Targeted Therapy Group, Division of Cancer Sciences, Faculty of Biology Medicine and Health, The University of Manchester, Manchester, UK; b Bioinformatics Core Facility, Michael Smith Building, The University of Manchester, Manchester, UK; c Mass and Flow Cytometry Core Facility, Cancer Research UK Manchester Institute, The University of Manchester, Manchester, UK; d Scientific Computing Core Facility, Cancer Research UK Manchester Institute, The University of Manchester, Manchester, UK; e Nuffield Department of Surgical Sciences, John Radcliffe Hospital, University of Oxford, Oxford, UK; f Patrick G. Johnston Centre for Cancer Research, Queen’s University of Belfast, Belfast, UK

**Keywords:** Radiotherapy, immunotherapy, Tregs, immune Checkpoints

## Abstract

Despite breakthroughs in immune checkpoint inhibitors (ICI), the majority of tumors, including those poorly infiltrated by CD8+ T cells or heavily infiltrated by immunosuppressive immune effector cells, are unlikely to result in clinically meaningful tumor responses. Radiation therapy (RT) has been combined with ICI to potentially overcome this resistance and improve response rates but reported clinical trial results have thus far been disappointing. Novel approaches are required to overcome this resistance and reprogram the immunosuppressive tumor microenvironment (TME) and address this major unmet clinical need. Using diverse preclinical tumor models of prostate and bladder cancer, including an autochthonous prostate tumor (Pten^−/−^/trp53^−/−^) that respond poorly to radiation therapy (RT) and anti-PD-L1 combinations, the key drivers of this resistance within the TME were profiled and used to develop rationalized combination therapies that simultaneously enhance activation of anti-cancer T cell responses and reprogram the immunosuppressive TME. The addition of anti-CD40mAb to RT resulted in an increase in IFN-y signaling, activation of Th−1 pathways with an increased infiltration of CD8+ T-cells and regulatory T-cells with associated activation of the CTLA−4 signaling pathway in the TME. Anti-CTLA−4mAb in combination with RT further reprogrammed the immunosuppressive TME, resulting in durable, long-term tumor control. Our data provide novel insights into the underlying mechanisms of the immunosuppressive TME that result in resistance to RT and anti-PD−1 inhibitors and inform therapeutic approaches to reprogramming the immune contexture in the TME to potentially improve tumor responses and clinical outcomes.

## Introduction

Immune checkpoint inhibitors (ICIs), such as αPD−1/PD-L1 or αCTLA−4 therapy have achieved “breakthrough cancer therapy” status as a result of durable tumor remissions seen in a number of metastatic cancers including melanoma, bladder, and lung cancers. Despite these successes, only the minority of cancer patients respond to ICI^1^ and in common cancers, such as prostate and breast cancers, ICIs have failed to make a significant clinical impact^2^. Radiation therapy (RT) is a highly effective cancer treatment delivered to approximately 40% of those cured of their disease. However, many patients will subsequently experience local and systemic recurrence and ultimately die of metastatic cancer^3,4^. In addition to direct tumor cytotoxicity, RT is now recognized to stimulate immune-mediated effects. These may include enhanced immunogenicity via upregulation of MHC-I in tumor cells,^5,6^ induction of immunogenic cell death, dendritic cell (DC) maturation, recruitment of tumor antigen-specific T-cells, and stimulation of type−1 IFN signaling^7,8^. The immunostimulatory effects of RT can potentially augment activation of tumor-specific T cells and provide a strong rationale for the combination of RT with ICI to enhance the generation of anti-cancer immunity^9,10^. However, whilst these and other pre-clinical tumor models have demonstrated promising results with RT and ICI combinations, the reported clinical trial results have been disappointing with little evidence for enhanced anti-tumor immune responses and subsequent low overall tumor response rates^11,12^.

A key driver of therapeutic resistance to RT and ICI combinations is thought to be the immunosuppressive tumor microenvironment (TME). Tumors displaying an immune landscape dominated by immunosuppressive cells including myeloid-derived populations, granulocytes, and regulatory T cells (T_reg_), are typically radioresistant and fail to respond to ICI^13^. In these tumors, the immunomodulatory effects of RT can also act to amplify immunosuppressive networks. RT can induce chemotactic signals leading to the recruitment of immunosuppressive myeloid cells, tumor associated macrophages (TAMs), expansion of Tregs^14^ and cause up-regulation of co-inhibitory ligands, such as PD-L1^9^, all of which can restrain anti-tumor efficacy. Consequently, there is an urgent unmet clinical need to develop therapeutic strategies that can potentially overcome tumor-related extrinsic resistance and augment the generation of anti-cancer immunity following RT. Therapeutic strategies to deplete, repolarize, or reprogram immunosuppressive cells within the TME are therefore attractive, in order to overcome environmental resistance and enhance responses in tumors that are resistant to RT and ICI.

In this study, we have profiled key drivers of resistance within the TME of diverse tumor types (prostate, bladder), that are known to lack clinical responsiveness to RT and ICI and have then used this data to develop rationalized combination therapies that simultaneously enhance activation of anti-cancer T cell responses and reprogram the immunosuppressive TME. We demonstrate that in tumors lacking T-cell infiltration and poorly responsive to fractionated RT, combination with an agonistic αCD40 monoclonal antibody (mAb) results in environmental reprogramming characterized by an increase in IFN-y signaling, activation of Th−1 pathways and increased infiltration of CD8+ T-cells in to the TME, which leads to enhanced tumor control over that observed with RT and αPD−1 combinations. Concomitantly, RT and αCD40mAb combination therapy resulted in an increase in the infiltration of Tregs and activation of the CTLA−4 signaling pathway within the TME. Administration of αCTLA−4mAb to RT and αCD40mAb therapy was able to overcome T-reg mediated immune-suppression resulting in an increased ratio of cytotoxic T-cells, tumor rejection, and development of long-term immunity. In conclusion, our data provide a mechanistic rationale for translating such potentially effective combination approaches into the clinic of common solid cancers that are therapeutically resistant to RT and ICIs.

## Materials and methods

### Cell lines

Murine prostate cancer cells (DVL3) were generated from tumors derived from the dorsal, ventral and lateral prostate lobes of a *Pten*^*−/−*^*/trp53*^*−/−*^ Pb-Cre4 mouse as previously described^15^. DVL3 cell lines were maintained in RPMI media supplemented with 10% FBS, L-glutamine and 10 nM DHT. The TRAMP-C1 murine prostate carcinoma cells were purchased from ATCC and maintained in DMEM high glucose medium, supplemented with 4 mM L-glutamine, 5% FBS, 5% Nu Serum (Corning, Bedford), 0.005 mg/mL of bovine insulin, and 10 nM dehydroisoandrosterone (Sigma, UK). The MB49 bladder cancer cells were purchased commercially and cultured in RPMI media containing 10% FBS.

### In vitro *clonogenic assay*

*In vitro* clonogenic assay was performed to assess the radiosensitivity and the full method can be accessed from the supplementary methods section.

### Tumor models

All animal experiments were performed under a United Kingdom Home Office License held at the CRUK Manchester Institute, University of Manchester (PCC943F76). Prior to each *in vivo* experiment, cells were screened for mycoplasma contamination and mouse hepatitis virus (MHV) at the Molecular Biology Core Facility (CRUK Manchester Institute). Mice were housed on a 12/12 light/dark cycle and were given filtered water and fed *ad libitum*. C57BL/6 male mice (6–8 weeks old) were inoculated subcutaneously at either the supra-spinal or flank position with either (5 × 10^6^) TRAMP-C1 or (1 × 10^6^) DVL3 cells, or (1 × 10^6^) MB49 cells under light-general anesthetic using isoflurane and oxygen gaseous mix in accordance with project license and home office regulations. Each cohort contained at least 4–5 mice housed in a single cage, based on power calculations from pilot studies. Tumor volume was measured using calipers as length × (width)^2^/2.

### Tumor therapy

Mice were randomized to treatment groups around 5–6 weeks post-cell inoculation when the tumor volume was at least 50–100 mm^3^. Irradiation was performed using our previously described method^16^ or using the new setup as described here forth. In brief, tumor-bearing mice were placed in a lead jig and shielded with just the tumors exposed. Mice were treated with X-ray top-down operating at 50KV, 10 mA with a 0.57AL filter, which gave a dose rate of 1.15 Gy per minute.

For immunotherapy studies, mice were treated with αPD-L1 (B7-HI) antibody i.p injection (clone 10F.9G2; BioXCell, USA) administered at 10 mg/kg, three times per week over a 2-week period or with isotype control Rat IgG2b.κb (BioXcell, USA) administered i.p, 3 times per week. Mice were treated with αCD40mAb (clone 3/23), a kind gift from Professor Martin Glennie, University of Southampton and/or commercially purchased from Biolegend, UK. The αCD40 antibody was administered (i.p) at a total dose of 500 µg, and the dose schedule of αCD40mAb was based on previous published investigations^17,18^.

Mice which received αCD40mAb sequentially received antibody on days 7 (200 µg), 10 (100 µg), 13 (100 µg) & 17 (100 µg), respectively. Mice were also treated with αCTLA−4 antibody (Clone 9D9; BioXcell, USA) sequentially on days 7, 10, 13 & 17, respectively; 200 µg per animal (i.p) or as indicated in the figure legends. Administration of FTY−720 (Fingolimod; Enzo Life Sciences, UK) commenced day prior to the start of RT and was delivered by oral gavage at a dose of 25 µg/mouse in a dosing volume of 200 µl. Subsequent daily administration was continued for 30 days (after the start of RT) at a dose of 5 µg/mouse in a dosing volume of 100 µl as previously described^19^.

For tumor rechallenge experiments, long-term surviving (LTS) mice were implanted contralaterally with DVL3 cells at least 90 days after therapy. Additional control mice were implanted at the same time to confirm tumor growth and study end point. Sample preparation for *ex viv*o processing of tissues for mass cytometry and immunohistochemistry is available in the supplementary methods section.

### Mass cytometry

The mass cytometry was done at the Flow Cytometry Core facility at the CRUK MI; the full methods is available in the supplementary methods section and in Table S1. Plotting and statistical analysis was done using R Statistical software and the result can be accessed using https://figshare.com/s/b27115e18ec570d755a7

### Immunohistochemistry

Immunohistochemistry was done both manually on the bench or on the Leica bond Rx platform and described in the supplementary methods section. All antibodies, source and concentration used for both multiplex and single plex immunohistochemistry have been listed in Table S2.

### Image acquisition and analysis

All chromagen slides were scanned digitally using Leica SCN−4000 slide scanner (Leica Microsystems) and the multiplex slides were scanned using either the Versa Slide scanner (Leica Micro Biosystems, UK) or the VS−120 slide scanner (Olympus). Image analysis and quantification was performed at the CRUK Manchester institute imaging facility using methods as described using the methods in the supplementary section.

### RNA extraction for nanostring and RNA seq analysis

RNA was extracted from FFPE mouse tumor tissue using the commercial kit from Norgen using their standard protocol on samples excised on day 15. This can be accessed in the supplementary methods section.

### Processing and normalization of NanoString data

Nanostring data was analyzed by Genomic Technology Core Facility (GTCF) at the University of Manchester and is available from the Array Express repository E-MTAB−11105. Negative and positive controls included in probe sets were used for background thresholding, and normalizing samples using the using nSolver analysis software (Version 4.0). Principal Components Analysis, and differential expression were calculated with DESeq2 v1.28.1^19^. K-means clustering was performed with R v4.0.3 and annotated heatmaps with Complex Heatmap v2.4.3 and cluster Profiler v3.16. DEGs were defined as passing *p* value of < 0.05. The resulting gene list was analyzed for pathway enrichment with IPA software using a reference gene list of the 770 genes on the Nanostring panel. The specific signature for IFN-y, MDSCs, Macrophages and DCs were derived from the literature or from the Nanostring Pan Cancel Panel.

### Statistical analysis

Results were analyzed using GraphPad Prism. Data were analyzed via a Shapiro-Wilk normality test to confirm that groups were distributed normally and analyzed by Student’s t-test. Non-parametric data was analyzed via Mann–Whitney testing. When comparing more than two groups a one-way analysis of variance (ANOVA) followed by a Tukey’s or Bonferroni multiple comparisons test was used to detect significant differences between means of each treatment group. Data are described with standard error of the mean (SEM). To compare survival curves from in vivo experiments, Log-Rank Mantel – Cox tests were performed on Kaplan – Meier plots.

## Results

### RT has no impact on the proportion or activation state of T-cells

**Figure 1.**
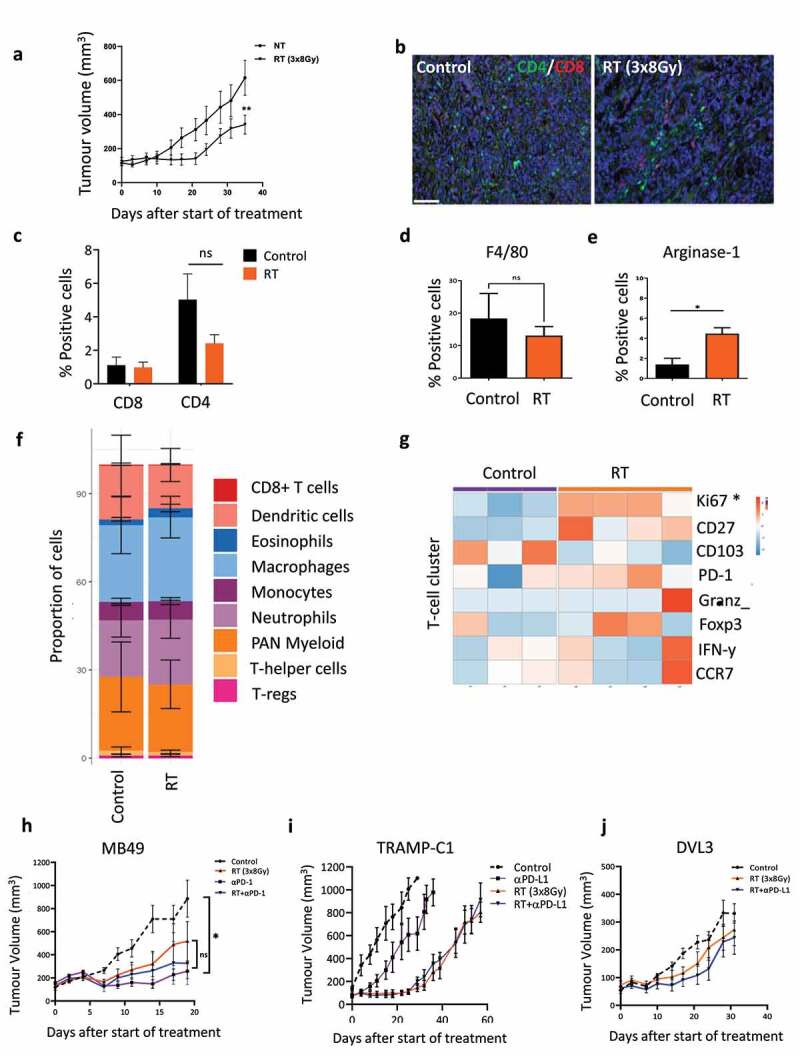
High dose fractionated RT had no significant impact on the tumor growth or on the proportion and activation state of T-cells. (a) the DVL3 tumors show therapeutic resistance *in* *vivo* when treated with high dose fractionated RT. DVL3 tumor bearing mice were randomized to treatment group and administered RT (3 fractions of 8Gy) on days 0,1 & 2. Tumor growth was measured over the 5 week period. Data represents mean tumour growth ± standard error (SEM) of at least 6–8 animals mice per treatment group. (b) Representative multiplex immunohistochemistry image of the tumor sections stained for CD4+ (Green) & CD8+ (Red) T-cells a week after RT in the DVL3 prostate tumor model (Scale bar 100 µM). (c) Quantification of intra-tumoral CD4+ and CD8+ T-cells in the TME of the DVL3 tumors following administration of fractionated (3x8Gy) RT demonstrating no significant increase in the T-cell numbers (*n*=4 mice per treatment group). (d,e) Quantification of intra-tumoral F4/80 and Arginase−1 expressing cells within the TME following administration of fractionated RT (3x8Gy fractions) in murine tumor model. * Denotes *p*<0.05 using unpaired T-test of (*n*=5 mice) per treatment group. (f) Frequency of tumor-infiltrating immune cells in DVL3 tumors at day 7 post-treatment, as measured by mass cytometry of *n*=3–4 mice per treatment group demonstrating that the tumors are heavily infiltrated with myeloid cells and have a low proportion of T-cells. (g) Hierarchical clustering and heatmap representing the mean intensity expression of select surface and intracellular markers associated within T-cell functional states measured by CyTOF analysis from individual animals. (h–i), Combination of fractionated RT (3x8Gy) and blockade of the PD−1/PD-L1 axis does not lead to long-term tumor control in the MB49, TRAMP-C1 and DVL3 tumor models. Both αPD−1 & αPD-L1 antibodies was dosed at 10 mg/kg, 3 times a week. Data represents tumor growth from start of treatment with at least *n*=4–5 mice per treatment group. *Denotes significant difference (*p*<0.05) using ANOVA and multiple comparison test.

Initially in order to better understand the potential local environmental drivers of therapeutic resistance to RT, we profiled the TME in immunosuppressive tumors lacking endogenous T-cells, using multiplex-IHC, flow cytometry and mass cytometry (CyTOF) in the less radiosensitive prostate DVL3 (Pten^−/−^/P53^−/−^) tumor model (Figure 1a and Figure S1a). Fractionated (3×8 Gy) RT failed to significantly increase the proportion of both CD8+ and CD4+ T-cells measured using multiplex IHC at day 7 (Figure 1b,c). In contrast, administration of fractionated RT resulted in a significant increase in the expression of Arginase−1, a marker expressed by tumor associated immunosuppressive macrophages (Figure 1d,e and Figure S1b). CyTOF analysis for deeper immune profile further confirmed that there were no significant changes in the overall proportion of T-cells, or other immune cell populations, after RT (Figure 1f and Figure S1c).

We next investigated whether the activation phenotype of tumor infiltrating T-cells was altered following RT from the CyTOF analysis. Using hierarchical clustering for surface and intracellular proteins, indicative of the T-cell functional state, we observed that expression of proteins associated with activation of T-cell function such as granzyme B, CCR7 and IFN-y showed no significant changes after RT, and no notable differences were observed in markers associated with T-cell dysfunction, such as PD−1 and Foxp−3 (Figure 1g and Figure S1d). The expression of surface molecules specifically on CD8 cytotoxic T-cells, which are known to be required for eradication of tumor, and dendritic cells (DC) were therefore investigated. Hierarchical clustering for surface and intracellular proteins revealed a decrease in CD103 expression and an increase in expression of CD27 on cytotoxic T-cell cluster; however, no changes were observed in the expression level of PD−1, IFN-y and CCR7 (Figure S1d). In the DC cluster, expression of IFN-y was increased in tumors, which received RT (Figure S1e). We also looked at MHC-II expressions for macrophages, which showed an overall increase in the positive proportion a week after RT administration (Figure S1f).

To establish whether targeting the PD−1/PD-L1 axis could overcome immunological resistance, we investigated the efficacy of administration of αPD-L1 therapy in the less radiosensitive prostate, and αPD−1 therapy^9,19^ in bladder tumor models as monotherapy or in combination with RT (Figure 1h,i, Figure S2). In the MB49 bladder tumors, administration of αPD−1 therapy as a single agent resulted in a marginal growth delay (Figure 1h). However, the administration of αPD-L1 therapy failed to significantly delay tumor growth in the TRAMP-C1 and DVL3 tumors (Figure 1i,j and Figure S2). In all the three tumor models targeting the PD−1/PD-L1 axis in combination with RT resulted in no significant growth delay or long-term clearance compared to monotherapy treatment alone (Figure 1h–j).

### Targeting CD40 induces reprogramming of tumor microenvironment that drives immune activation and T cell infiltration

In tumors that are refractory to checkpoint blockade, targeting costimulatory pathways to enhance T cell priming may be an alternative effective strategy to overcome such therapeutic resistance. In the DVL3 model, the administration of fractionated RT upregulated CD40 expression in tumors compared to control non-treated animals (Figure S3). Taken together with our previous published work showing agonistic anti-CD40 antibody could synergize with radiation to elicit DC-dependent priming of T cell immunity^18,20^ this provided a rationale for targeting CD40 to enhance T-cell priming in our radioresistant, anti-PD−1/PD-L1 refractory models.

**Figure 2.**
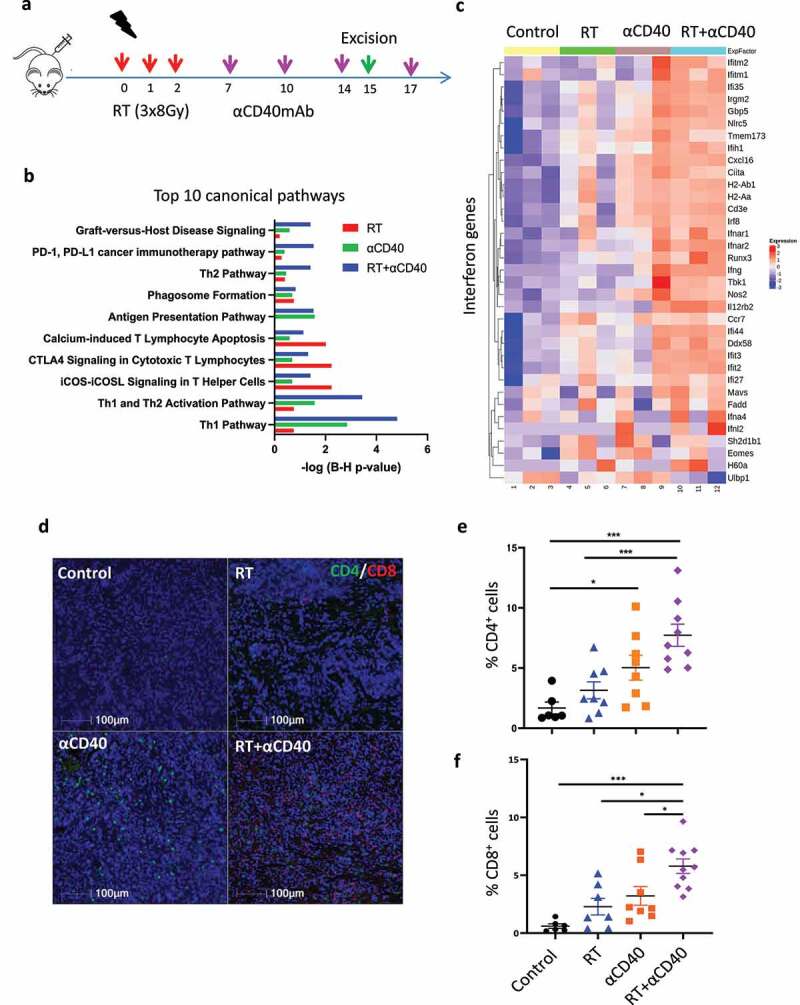
Targeting CD40 drives immune activation and T-cell infiltration. (a) Schema of study design for immune profiling of the DVL3 tumours following administration of αCD40 and RT alone or in combination. A cohort of mice were culled on day 15 for immune profiling and ex vivo analysis. Another cohort of mice were allocated to therapy arms and were kept until mice reached the study endpoint. C57Bl/6 male mice were implanted with (1 X10^^6^) DVL3 cells or (5 X10^^6^) TRAMP-C1 cells in the supra spinal or the right flank position. Once the tumors were established (4–6 weeks post-cell inoculation), mice were randomized and treated with fractionated RT (8Gy in 3 fractions delivered on consecutive days). αCD40 antibody was administered sequentially from day 7, and subsequently on days 10, 14 & 17. Excision samples were taken at the indicated time point Day 15. (b) Nano-string® gene expression analysis in the DVL3 tumors following RT and αCD40 therapy to re-program the TME. Top canonical pathways from the downstream analysis of the log2 transformed normalized mRNA count using IPA software. Graph shows significantly enriched canonical pathways identified from IPA® across different treatment conditions compared to control treated tumors. The pathway shown are log-p value with B-H correction applied to account for false discovery rates (FDRs). The Th1 pathway and the Th1 and Th2 activation pathway were amongst the top pathway enriched in the combination treated tumors. (c) Modular heat map of interferon genes showing increase in expression in the combination treated tumors and in monotherapy group alone (*n*=3 mice per group). (d) Representative multiplex IHC images for CD4+ and CD8+ T-cells in the DVL3 tumors. Mice were treated as schematic, and on day 15, tumor samples were excised, and sections evaluated for the changes within the T-cell compartment. αCD40mAb in combination with RT resulted in a significant increase in both CD8+ and CD4+ T-cells in the DVL3 tumors compared to RT treated tumors. (e) Quantification of intra-tumoral CD4+ T-cells within the TME in the DVL3 tumors showing an increase in overall proportion of CD4+ T-cells in αCD40 and combination treated animals. (f) Quantification of intra-tumoral CD8+ T-cells in the RT and αCD40 combination treated animals demonstrating a significant increase in the proportion of cytotoxic-T-cells compared to all three treatment groups. Multiplex IHC data represents the mean + SEM of *n*=5–8 animals per treatment group from 2 independent experiments. * Denotes *p*<0.05; **, *p*<0.005, *** *p*<0.0005 using ANNOVA and multiple comparison correction applied.

We therefore investigated whether the addition of αCD40mAb sequentially after RT was able to recalibrate the TME to favor T-cell activation in the highly immunosuppressive and less radiosensitive DVL3 tumor model. Using the Nanostring (n-Counter®) Pan Cancer immune panel, we investigated differential gene expression following treatment with αCD40 or RT alone and in combination (Figure 2 and Figure S4). Nanostring gene expression profiling was undertaken in serial sections from FFPE blocks excised from samples taken day 15 post the start of RT matching the immunohistochemistry samples (as shown in the schema, Figure 2a). The Nanostring gene expression analysis identified over 350 genes upregulated in both αCD40 alone and in combination treated tumors, and greater than 220 genes upregulated in RT treated tumors using a cut off, *p* value < 0.05 (Figure S4a-c). The Gene Ontology (GO) from the gene expression profiling identified a number of biological processes in αCD40mAb and RT treated tumors with the greatest changes in biological processes observed in the combination treated tumors (Figure S4d).

To identify predictive pathways associated with differential gene expression, downstream analysis was undertaken using the Ingenuity Pathway Analysis tool IPA® (Figure 2b). Amongst the top 5 predicted pathways, Th1 and Th2 activation, antigen presentation, and iCOS-iCOSL signaling in T Helper Cells were enriched in the combination treated tumors. Likewise, the αCD40mAb resulted in activation of Th1 and Th2 and antigen presentation pathways. In contrast, the top canonical pathways in RT treated tumors included iCOS-iCOSL signaling in T Helper cells and CTLA−4 signaling in cytotoxic T lymphocytes (Figure 2b). The differential expressed genes were then analyzed using complex modular heat maps for specific gene sets associated with interferon (IFN) signaling, DC activation, macrophage re-programming and myeloid cells. The genes associated with IFN-y signaling were highly expressed in the RT and αCD40mAb combination treated tumors (Figure 2c), which corroborated the significant increase in intra-tumoral CD8+ T-cells observed using multiplex IHC in matched tumor samples (Figure 2d). Administration of αCD40mAb resulted in a significant increase in CD4+T-cells compared to control animals (*p* < 0.05) both as monotherapy and in the combination with RT (*p* < 0.005, Figure 2e). Sequential administration of αCD40mAb in combination with RT led to a significant increase in CD8+ T-cells (*p* < 0.005) compared to control tumors and RT groups (*p* < 0.05) (Figure 2f).

**Figure 3.**
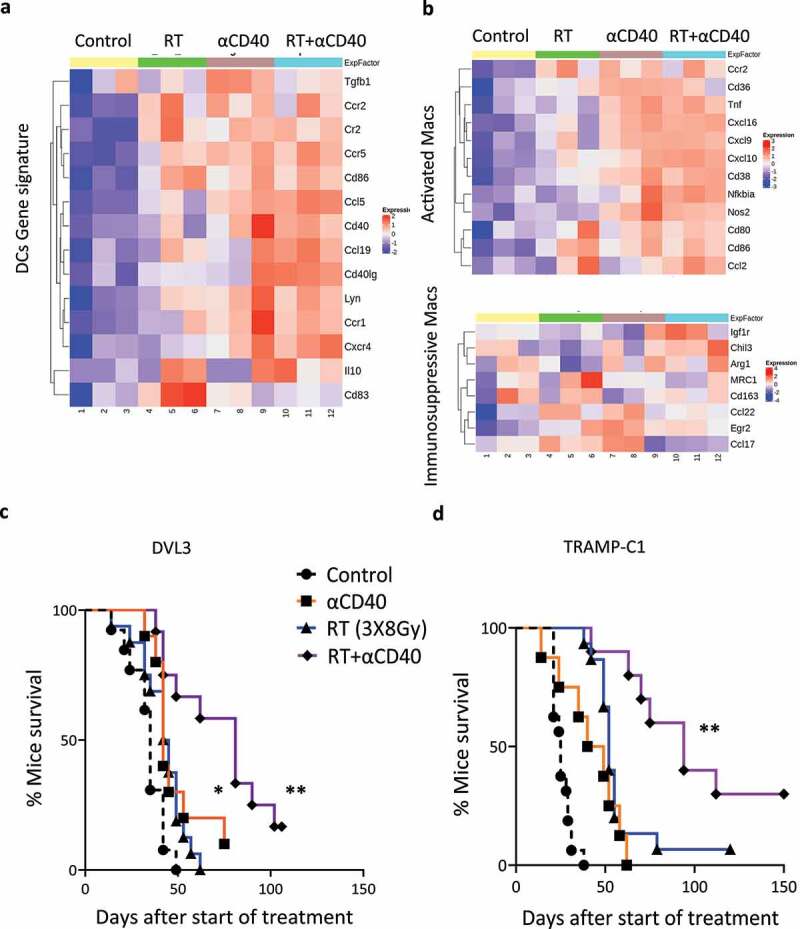
αCD40mAb in combination with RT induces re-programming of tumor microenvironment (TME) and enhances tumor control in radioresistant tumor models. (a) Modular heat map of genes associated with dendritic cells (DCs) showing high expression in both αCD40 and combination (RT+αCD40) treated tumors. (b) Heat map of genes associated with classical pro-inflammatory and immunosuppressive macrophages demonstrating an increase in expression for genes associated with pro-inflammatory macrophages in combination (RT+ αCD40) treated tumors. Heatmap values are the scaled log2 transformed data from (*n*=3) samples per treatment group. (c) Kaplan Meir survival demonstrating improved survival and tumor clearance in the combination treatment arm when the αCD40 was administered sequential to RT starting on day 7 in the DVL3 tumors. (d) Kaplan-Meir survival in the TRAMP-C1 tumor model demonstrating improved survival in the combination treated group. Data represents two independent experiments with at least *n*=8–10 mice per treatment group. **, *p*<0.01, *p<0.05, Log-rank (Mantel-Cox) test.

The heat map for DC activation showed increased expression for CD86, Ccl5, CD40, Ccl19, Ccr1 and Cxcr4 in both αCD40mAb and RT and αCD40mAb combination treated tumors (Figure 3a). Macrophages showed an increase in the expression of genes associated with classical activation following treatment with αCD40 alone, or in combination with RT (Figure 3b). Gene signature associated with MDSCs also increased in both αCD40 and combination treated tumors (Figure S5a). This is correlated with a significant increase in intra-tumoral PAN myeloid cells (CD11b+) following administration of αCD40mAb both as monotherapy and in combination with fractionated RT; and granulocytic myeloid cells (CD11b+Ly6G+) after αCD40 treatment alone, compared to non-treated tumors (Figure S5b–d).

In parallel, we have also investigated the systemic immune effects of αCD40mAb alone or in combination with RT in the blood of tumor-bearing mice (Figure S6). In both animals treated with αCD40mAb alone or the combination therapy, an increase in CD40L expression was observed on CD4+ T-cells, but not on CD8+ T-cells (Figure S6b,c *p* = 0.01, *p* = 0.005). The increase in CD40L expression on CD4+ T-cells correlated with an increase in the proportion of both CD4+ and CD8+ T-cells in the blood of mice given combination therapy (Figure S6d,e *p* = 0.008, *p* = 0.0043 vs RT respectively).

### αCD40mAb in combination with RT enhances tumor control in radioresistant tumors

Next, we investigated whether the addition of αCD40mAb to RT enhanced therapeutic benefit. When the αCD40mAb was given sequentially after RT in DVL3 tumors, it resulted in improved tumor control compared to RT alone with around 20% long-term survivors (Figure 3c,d and Figure S7). αCD40mAb administered as monotherapy resulted in modest improvements in tumor control compared to non-treated animals in both the DVL3 and TRAMP-C1 tumor models (Figure 3c,d and Figure S7).

We also evaluated the effect of concurrent administration of αCD40mAb in combination with fractionated RT. Concurrent administration of αCD40mAb in combination with RT resulted in a marginal tumor growth delay compared to RT group alone (Figure S8a,b). The level of CD8+ T-cells in the combination treated group was comparable to αCD40mAb group alone (Figure S8c,d). Taken together with the TME profiling data, the sequential rather than concomitant administration of RT and αCD40mAb appears to result in both reprogramming of TME and enhanced tumor control. Finally, we investigated whether αCD40mAb could radio-sensitize these highly immunosuppressive tumors to low dose, fractionated RT (schema shown in Figure S9a). Administration of 2 Gy in 5 daily fractions (5×2 Gy) RT in combination with αCD40mAb resulted in no significant benefit compared to RT alone in both the TRAMP-C1 and radioresistant DVL3 tumors (Figure S9b,c).

### RT and αCD40 mAb combination therapy resulted in expression of immune check points and expansion of T-regs in the TME

**Figure 4.**
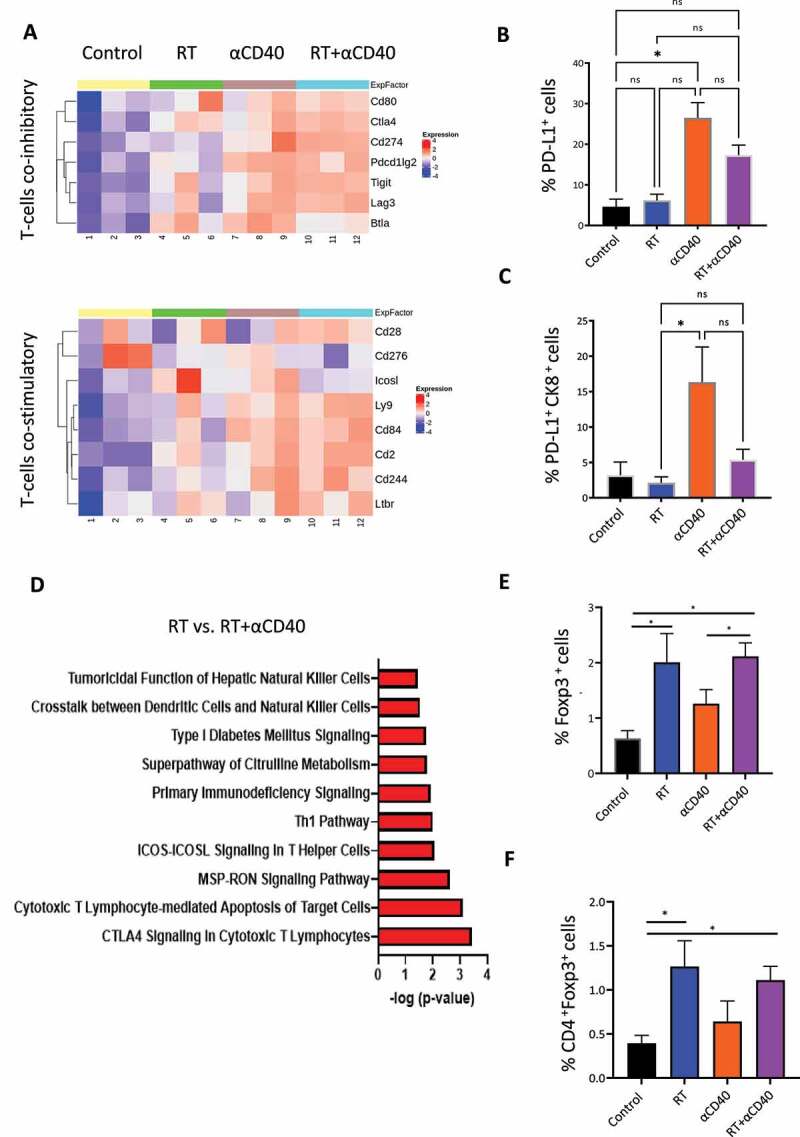
RT and αCD40 combination therapy resulted in an activation of CTLA−4 signaling and expansion of regulatory T-cells (Tregs). (a) Modular heat map for genes associated with co-inhibitory and co-stimulatory molecules expressed on immune cells analyzed using Nanostring (nCounter®) on excised FFPE tumors samples. Hierarchical clustering was performed on the scaled log2 transformed gene expression data (higher expression in red and low expression in light/dark blue). (b) Multiplex IHC staining looking at the expression of PD-L1 staining within the TME showing an overall increase in the αCD40 and the combination treatment group. Data represents mean ± SEM of at least 3–4 mice per time point. (c) Multiplex IHC staining looking at the expression of PD-L1 staining on tumor cells (CK8+) cells. Quantification of PD-L1 staining demonstrated no significant increase in PD-L1 expression on tumor cells (CK8+) after RT or in the combination (RT and αCD40) treatment. Data represents mean ± SEM of at least 3–4 mice per time point. (d) Top canonical pathways identified from the downstream analysis of the log2 transformed normalized mRNA count comparing DEGs in the combination group to RT treated tumors. The canonical pathway analysis identified activation of CTLA−4 signaling on cytotoxic T-lymphocytes. (e,f) Quantification of intra-tumoral Foxp3+ and CD4+Foxp3+ T-cells demonstrating a significant increase in the combination treatment group. Data represents the mean + SEM of *n*=5–8 animals per treatment group. * Denotes *p*<0.05 using ANOVA and multiple comparison test applied.

To evaluate whether the increase in intra-tumoral T-cells following RT and αCD40mAb therapy results in an increase in expression of genes associated with T-cell “activated” or “exhausted” functional states, we investigated the expression of T-cell co-inhibitory and costimulatory genes from the Nanostring sequencing dataset. The modular heat map showed that both PD-L1 (CD274) and CTLA−4 checkpoints were highly expressed in combination treated tumors (Figure 4a). Next, we validated the nanostring dataset using IHC to confirm an increase in PD-L1 protein expression in tumors treated with αCD40 (p < 0.05), which was not seen with RT alone or RT in combination with αCD40mAb (Figure 4b). We also investigated the co-expression of PD-L1 protein staining in tumor cells using cytokeratin (CK8) staining, and found that there was no significant increase in PD-L1 expression on tumor cells with RT alone or in the combination treated tumors compared to control tumors (Figure 4c and Figure S10a).

To identify predictive pathways associated with differential gene expression, downstream analysis was undertaken using the IPA® comparing differentially expressed genes from RT and combination treated tumors. By comparing the two treatment groups, the top pathway from the analysis identified CTLA−4 signaling on cytotoxic T lymphocytes being significantly enriched with a positive score (Figure 4d). To validate the gene expression findings, we investigated whether the higher expression of CTLA−4 resulted in the expansion of regulatory (CD4+ Foxp3+) T-cells, using multiplex immunohistochemistry (Figure S10b). Both RT and the RT and αCD40mAb combination treatment resulted in a significant increase in the proportion of Foxp3+ cells within the TME (*p* < 0.05 Figure 4e), as well as a significant increase in CD4+ and Foxp3+ cells (*p* < 0.05, Figure 4f).

### RT and αCD40 combination therapy drives tumor infiltrating T-cells and improved tumor control

**Figure 5.**
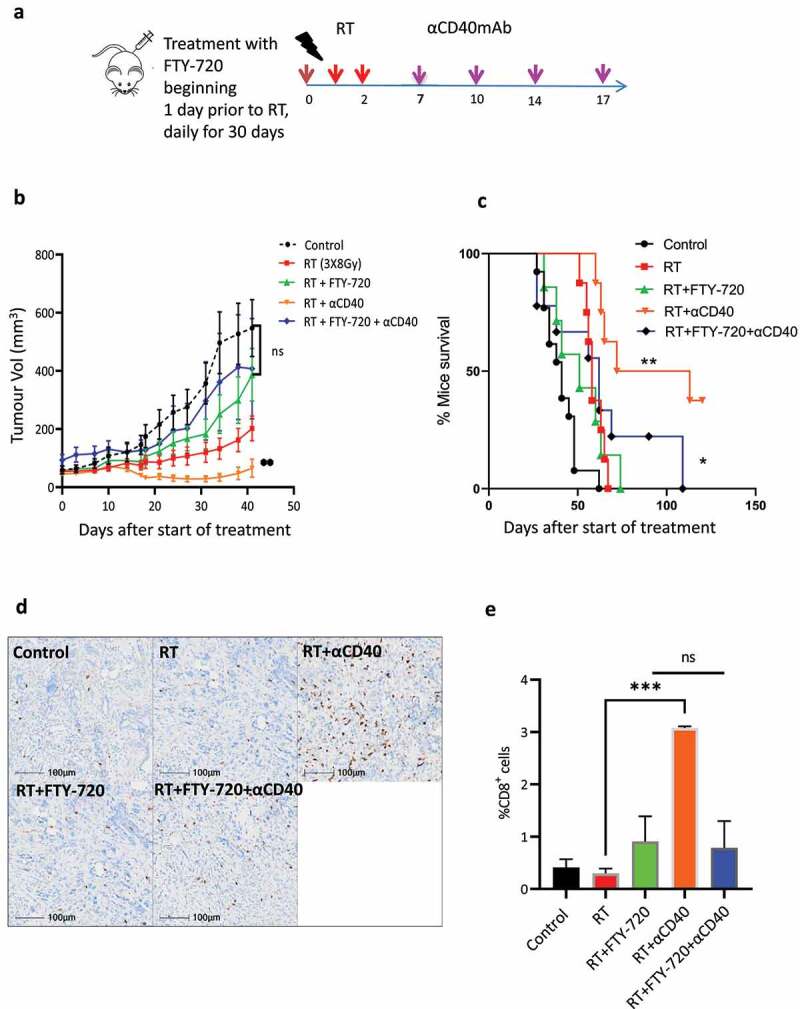
Tumor infiltrating T-cells contribute to the therapeutic efficacy following fractionated RT and αCD40mAb therapy. (a) Schema for *in* *vivo* study for evaluating the effect of FTY−720. DVL3 tumor bearing mice received RT delivered in 3 daily fractions of 8Gy in combination with sequential αCD40mAb administration dosed on days 7, 10, 14, & 17, respectively. A cohort of mice received FTY−720 (a sphingosine 1-phosphate receptor agonists) which prohibits T-cell emigration from lymphoid tissues. FTY−720 was dosed at 25ug/mouse for the first dose, and 5ug/mouse daily dosing for 4 weeks. (b) Mean tumor volume (MTV) showing the therapeutic efficacy of RT and αCD40mAb is reduced when mice are administered FTY−720 starting before RT. Data represents mean +SEM of at least 6–9 mice per treatment group. **Denotes statistical significance using ANOVA. (c) Kaplan Meir survival analysis demonstrating efficacy of RT and αCD40 therapy is reduced when mice were administered FTY−720 prior to therapy. *, *p*<0.05, **, *p*<0.01, Log-rank (Mantel-Cox) test. (d) Representative IHC images for CD8+ T-cells on tumors sections excised on day 15 post initiation of treatment demonstrating that FTY−720 blocks infiltration of CD8+ immune cells into the tumor from the draining lymph node. Scale bar = 100µM. (e) Quantification of IHC staining for CD8+ staining demonstrating that the proportion of CD8+ is significantly reduced in the tumor when mice were treated with FTY−720 prior to radiotherapy and in the RT/αCD40 combination treated animals. Data represents mean + SEM of *n*= 3 samples per treatment group. Scale bar = 100uM.

In order to investigate the potential contribution of infiltrating T-cells on tumor regression following combination therapy, FTY−720 (a sphingosine 1-phosphate receptor agonist) was used to impair T-cell emigration from lymphoid organs^21,22^. We have previously demonstrated that treatment with low-doses of FTY−720 had no effect on tumor growth despite substantially reducing the numbers of T-cell populations infiltrating into the tumor^19^. Administration of FTY−720 prior to RT, resulted in an accelerated tumor growth compared to RT treatment alone, but no overall difference in survival (Figure 5a–c). When FTY−720 was administered prior to RT and αCD40mAb combination it resulted in abrogation of the therapeutic effect of the treatment, and tumor growth was comparable to controls (Figure 5b,c). To evaluate whether the FTY−720 was blocking the infiltration of T-cells within the TME, we profiled tumor samples. Administration of FTY−720 prior to combination therapy resulted in comparable levels of CD8+ T-cell infiltrates to RT alone or the controls. As expected, in the absence of FTY−720, RT and αCD40mAb combination group had significantly higher proportion of CD8+ T-cell infiltrates, as shown before (p < 0.05, Figure 5d,e). These data imply that the RT and αCD40mAb combination results in an anti-tumor immune response leading to tumor infiltrating T-cells that appear to contribute to the tumor control, which is not observed with RT and ICI combinations.

### αCTLA−4 therapy overcomes T-reg suppression to enhance RT and αCD40mAb leading to long-term tumor control

As the profiling studies showed an increase in expression of both PD-L1 and CTLA−4 within the TME in response to combination therapy, we next investigated whether targeting these immune checkpoint pathways could further enhance long-term tumor control. The addition of αPD-L1 therapy to the combination of RT and αCD40 mAb had no significant survival benefit or long-term impact on tumor control in the DVL3 tumor model (Figure S10c,d). This data is in keeping with the earlier observation that DVL3 tumors are resistant to PD-L1 therapy (Figure S2).

**Figure 6.**
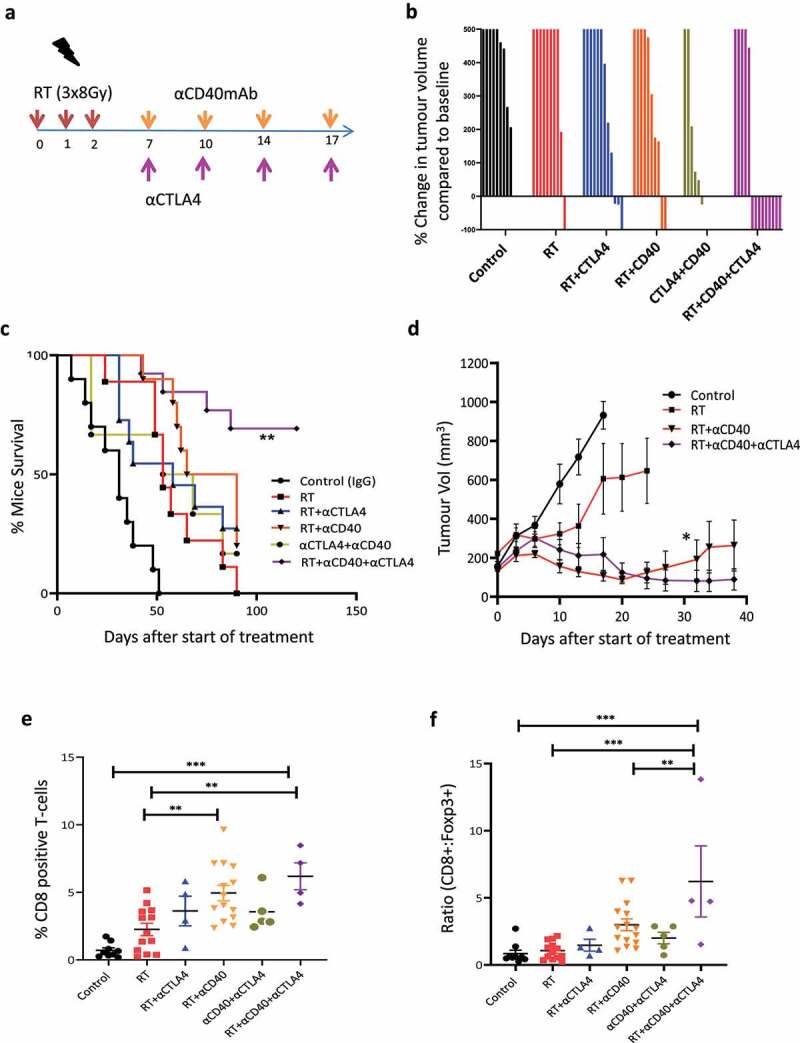
Administration of αCTLA−4mAb improves the therapeutic efficacy of RT and αCD40 combination treatment leading to long-term survival in prostate and bladder cancer models. (a) Schema of the study design for *in* *vivo* therapy experiment. (b) Waterfall plot showing the maximum percentage change in tumor volume from baseline in individually treated mice following treatment with αCTLA−4 antibody in combination with RT and αCD40 treatment in the DVL3 tumors. Each waterfall plot represents individual mice ranging from 6–14 animals per treatment group, from 2 independent experiments. (c) Kaplan-Meir survival curve demonstrating improved survival in the triple combination group with tumor control in majority of mice in the DVL3 tumors. Data presented from 2 independent experiments with at least 6 animals. C57Bl/6 male mice were implanted with (1 ×10^6^) DVL3 in the supraspinal position. Once the tumors were established (4–6 weeks post cell inoculation), mice were randomized and administered 24Gy RT delivered in 3 daily fractions of 8Gy as per schema. αCD40mAb was administered sequentially from day 7, and subsequently on days 10,14 & 17. αCTLA−4 therapy was administered as per the schema on days as indicated**, *p*<0.01, *p<0.05, Log-rank (Mantel-Cox) test. (d) Mean tumor volume demonstrating enhanced tumor control in the murine bladder (MB49) tumor model following administration of αCTLA−4 antibody in combination with RT and αCD40. (1X10^6^) MB49 cells were inoculated in C57BL/6 mice. 2 weeks post inoculation mice were randomized to treatment group and treated as per the schema A. Individual tumor growth data is shown in the supplementary figure -S11. * Denotes *p*<0.05 in the triple combination group. (e) Quantification of intra-tumoral T-cells within the TME in the DVL3 tumors showing an overall significant increase in the proportion of CD8+ T-cells in both double and triple combination treated animals. Mice were treated as schema, and on day 15, tumor samples were excised from all the treatment groups. Tumor sections were evaluated for the changes within the T-cell compartment. αCD40mAb in combination with RT resulted in a significant increase in both CD8+ in the DVL3 tumors compared to RT treated tumors. Addition of αCTLA−4 to RT and αCD40 therapy resulted in a significant increase in intra-tumoral CD8+T cells compared to RT and non-treated controls. ** denotes *p*<0.01 using ANNOVA and multiple comparison correction applied. (f) Quantification of the CD8: Treg ratio in the treated mice showing a significant increase in the ratio in triple combination group compared to tumor bearing mice which were treated with RT and αCD40 combination therapy or RT alone. ** denotes *p*<0.01; ***p<0.001 using ANNOVA and multiple comparison correction applied.

Given that the activation of CTLA−4 signaling and expansion of regulatory T-cells was observed in the DVL3 tumors following RT and αCD40mAb combination therapy we therefore hypothesized that the addition αCTLA−4mAb would abrogate the immunosuppressive CTLA−4 signaling and result in enhanced tumor control (treatment schema shown in Figure 6a). The combination of RT and αCTLA−4mAb resulted in a comparable level of tumor control as RT alone as shown in waterfall plots (Figure 6b) and Kaplan–Meier survival curves (median survival 58 vs 53 days, respectively) (Figure 6c) in the DVL3 tumor model. αCD40mAb and αCTLA−4 combination had similar levels of tumor control and failed to deliver long-term survival. In contrast, administration of both αCD40mAb and αCTLA−4mAb sequentially after RT resulted in tumor control in the majority of mice and improved the overall survival compared to RT and αCD40mAb treatment group (p = 0.04, mantel-Cox test) and RT treatment (p = 0.0004, mantel-Cox test) (Figure 6b,c). We also investigated the effect of this triple combination of RT and αCD40mAb and αCTLA−4mAb in the MB49 bladder tumor model. Administration of αCD40mAb/αCTLA−4mAb sequentially after RT resulted in significant growth delay compared to RT or RT/αCD40 combination treatment (Figure 6d and Figure S11a).

To evaluate long-term anti-tumor immunity, mice from the triple combination group with complete response were re-challenged with the DVL3 tumor cells in the left-flank and compared to naïve animals. Of the long-term surviving mice, 75% of the mice rejected tumors on the left flank, which indicated the development of long-term durable and systemic anti-tumor immunity following the addition of αCTLA−4 therapy to the RT and αCD40mAb combination (Figure S11b).

To probe the microenvironmental changes, we profiled the TME following triple combination therapy during treatment. The triple combination resulted in a significant increase in the proportion of intra-tumoral CD8+ T-cells compared to RT and non-treated tumors (*p* < 0.001, Figure 6e). The addition of α-CTLA−4mAb to RT and αCD40mAb therapy also resulted in a significant increase in the ratio of CD8+: Foxp3+ cells compared to RT and αCD40mAb group (*p* < 0.05) and to RT alone and control tumor samples (*p* < 0.001, Figure 6f). In summary, only the addition of αCTLA−4mAb to the combination of RT and αCD40mAb led to long-term tumor control, indicative of the importance of overcoming this immunosuppressive signaling pathway.

## Discussion

In this study, we have investigated the underlying mechanisms of resistance to RT and ICI combinations and used these mechanistic data to inform approaches to overcome this therapeutic resistance and enhance tumor control. The major findings to emerge from this study were that the administration of αCD40mAb in combination with RT resulted in an increase in infiltration of cytotoxic T-cells, activation of genes associated with DC maturation, an increase in IFN-y signaling and activation of Th1 pathway that resulted in enhanced tumor control. Importantly RT/αCD40mAb combination therapy also resulted in an increase in infiltration of regulatory T-cells (Tregs), activation of CTLA−4 signaling on T-cells, and an increased expression of PD-L1 in macrophages, but not in tumor cells within the TME. Sequential administration of αCTLA−4mAb to RT/αCD40mAb combination therapy was able to reverse these potentially immunosuppressive signals, and resulted in an increase in both intra-tumoral CD8 T cells and the CD8: T-reg ratio, leading to durable long-term tumor control.

A number of preclinical studies have previously reported durable anti-tumor immune responses, leading to long-term tumor control by combining 3 × 8 Gy RT with ICI using either αPD−1/PD-L1mAb^23^, or αCTLA−4 mAb^24,25^. However, the most effective responses are generally observed in tumors that have higher levels of endogenous T cell infiltration^17,18^. In contrast to these studies, in our tumor models, which are poorly infiltrated by T cells, and resistant to RT and αPD−1/PD-L1, we observed an increase in Ki−67 expression, suggesting an increase in T-cell proliferation, but no effect on either the proportion of CD4+ or CD8+ T-cells nor on IFN-y production by CD8+ T-cells. Given that higher doses of RT have been suggested to be immunostimulatory, the increased T-cell proliferation may be due to *in situ* co-stimulation provided by tumor resident APCs. Furthermore, the impact of RT dose on immune modulation and ability to enhance immunotherapy in tumors lacking T cell infiltration may be critical, but remains poorly understood. A single fraction of low-dose RT (1 Gy) has been shown to improve responses to checkpoint blockade in tumors with an immune “desert” phenotype^26^. Similarly, agonistic αCD40mAb in combination with a single fraction of 5 Gy RT has previously been evaluated in the TRAMP-C1 murine prostate model and shown to enhance efficacy of checkpoint blockade^27^. In our tumor model, low dose fractionated RT (5×2 Gy) in combination with αCD40mAb failed to deliver tumor control in contrast to 3 × 8 Gy, which resulted in tumor clearance and systemic immunity in over 65% of mice. Our findings in the prostate DVL3 model are in keeping with reports from other murine models, demonstrating the ability of αCD40 mAb to license APCs to effectively initiate a cytotoxic T-cell response when administered in combination with RT^18,28^.

RT had no effect on the number of DCs or T-cells within irradiated tumor at day 7. By day 15, there was a higher expression of transcripts associated with APC activation (CD86, CCR5, CCR2) within the TME. RT has been shown to trigger innate immune signaling pathways associated with enhanced recruitment, maturation, and functional activation of DC, both within the TME and draining lymph nodes^29^. RT induced tumor cell death may also enhance the pool of tumor antigen available^30^. αCD40mAb is known to augment DC licensing and cross-presentation of tumor antigen^31,32^, and we have previously reported DC’s to be critical to the generation of T-cell immunity in response to the combination of RT and αCD40 mAb^18^. In keeping with this observation in the RT and αCD40 combination treated mice, we found a culmination of APC activation and an increase in the numbers of both CD4 and CD8+ T-cells both systemically and within the TME, during the peak of tumor regression. Interestingly, the increase in the number of TILs and the efficacy of RT and αCD40 mAb was abrogated in mice treated with FTY−720 which blocks the emigration of T-cells from the lymph nodes. These results suggest that newly-primed, infiltrating effector CD8 T-cells are required to mediate anti-tumor immunity in response to this combination therapy, rather than relying on resident T-cells, which may also contribute to tumor regression^22^. FTY−720 has previously been shown to directly sensitize tumor cells to RT, including prostate cancer^33^. However, treatment with FTY−720 did not enhance the RT tumor control in our murine prostate models. Indeed, these prostate tumors appear to grow faster in FTY−720 treated mice which may reflect a role for such infiltrating tumor T-cells in delaying the initial tumor growth after inoculation, and when this is inhibited the tumor grows more rapidly.

Although CD40 agonists and ICI have different pharmacodynamic effects, both can lead to IFN-y signaling, which can result in upregulation of immune checkpoints within the TME^34,35^. αCD40mAb in combination with RT can sensitize immunologically cold-tumors to αPD-L1 therapy^36^. In our study, treatment with αCD40mAb alone, or in combination with RT, resulted in elevated expression of both CTLA−4, and PD-L1 checkpoint molecules within the TME. Interestingly, in the prostate cancer model, the PD-L1 expression was primarily restricted to macrophages and stromal areas and was not expressed in tumor cells. This is in keeping with previously published observations from other immunologically “cold” tumor models, such as pancreatic ductal adenocarcinoma (PDAC) that lack T cell infiltrates^35^. Although the significance of this observation with PD-L1 expression is currently unknown, it could be both tumor and immune contexture dependent and warrants further investigation. In the DVL3 model, the addition of αPD-L1 to RT and αCD40mAb combination therapy, failed to improve tumor control. In contrast, the addition of αCTLA−4mAb to RT and αCD40mAb treated mice resulted in sustained and durable anti-tumor immunity. αCTLA−4mAb has been shown to impair Treg function^37^, and mediate Treg depletion in murine tumors,^38^ although the dominant mechanisms of action may differ in human cancers^39^. In our models, administration of αCTLA−4mAb did not decrease Tregs within the TME, but did significantly increase the ratio of CD8: Treg, a predictive biomarker of response to cancer treatment including ICB and RT^39,40^. This likely reflects the significant increase in effector CD8 T-cells observed within the TME following triple combination therapy. Targeting CTLA−4 can attenuate T cell priming through co-inhibition of intrinsic signaling pathways^41,42^ and trans endocytosis of costimulatory molecules. Given that blockade of T-cell infiltration into the TME with FTY−720 abrogates the efficacy of combination therapy, it is possible that addition of αCTLA−4 mAb to RT and αCD40 mAb, primarily functions to increase priming of effector T cells within the lymph nodes.

Overcoming the immunosuppressive TME and enhancing tumor response to RT using immunomodulatory agents is likely to require targeting both innate and adaptive immune effector pathways. Here, we demonstrate that rationalized dual targeting of co-stimulatory and co-inhibitory immune checkpoints can enhance tumor control in combination with RT. Such combinations exploit distinct, but complementary mechanisms of action that augment the generation of T cell immunity. Targeting of more than one checkpoint using a combination of anti-PD−1 and anti-CTL4–4 therapy has been shown to enhance clinical outcomes in metastatic malignant melanoma in clinical trials^43^. The diversity and range of checkpoints explored in pre-clinical models and as monotherapy in the clinic, provides an opportunity to explore the synergistic activity of multiple combinations. However, such combinations are more likely to be effective in enhancing tumor control if guided by detailed mechanistic understanding of the suppressive networks operating within the TME. Successfully unlocking the potential of manipulating the tumor immune contexture to improve treatment response rates and outcomes in tumor resistance to RT and anti-PD-1 requires well-designed clinical trials with immunomodulatory agents that investigate the baseline immune contexture within the TME. Thus far hundreds of clinical trials have been set up with an over dependence on PD−1/PD-L1 blockade in combination with RT with largely disappointing results. Our data suggest that for tumors which are poorly infiltrated by T cells, future clinical trial designs should investigate alternative immunostimulatory agents in combination with alternative RT doses and fractionation aimed at reprogramming the TME to overcome therapeutic resistance to ICI.

## Supplementary Material

Supplemental Material
