# Supplementary material for: Reprogramming the immunosuppressive tumor microenvironment results in successful clearance of tumors resistant to radiation therapy and anti-PD-1/PD-L1
Source: Oncoimmunology. 2023 Jun 15;12(1):2223094. doi: 10.1080/2162402X.2023.2223094 (PMC10274532; doi:10.1080/2162402X.2023.2223094)
Supplement: Supplemental Material [file KONI_A_2223094_SM4288.zip › Supplemental Figure Legends.docx]

**Supplemental Figure Legends:**

**Figure-S1: A,** *Radiosensitivity* measurement of murine cancer cells (CT26, 4T1, DVL3 and TRAMPC1) using the in vitro clonogenic assay showing DVL3 tumor models are significantly less radiosensitive. The murine cancer cells (approx. 25-1600 cells) were seeded in 6 well plates and incubated in complete medium at 37°C for 4 hours. Plates were irradiated at increasing doses and incubated for 8-10 days. At least three independent experiments were carried out and the plating efficiency and survival fraction was calculated. Linear quadratic analysis was performed for this assay, results were reported as mean± SEM of n=3-4 experimental replicates. **B,** Representative IHC images for Arginase-1 staining in the TRAMP-C1 tumor model**. C,** Uniform Manifold Approximation and Projection (UMAP) plots displaying the relative abundance of different cell clusters as defined by unsupervised self-organizing map clustering (FlowSOM).  **D,** Hierarchical clustering and heap map of the mean intensity expression of surface and intracellular markers associated with cytotoxic (CD8) T-cells. **E**, Hierarchical clustering and heap map of the mean intensity expression of Dendritic cells (DCs) measured by mass cytometry. **F,** Administration of fractionated RT resulted in marginal increase in the proportion of MHC-II expressing macrophages. The CYTOF data was performed on 3 controls and 4 RT treated tumors.

**Figure-S2: Administration of αPD-L1 does not lead to tumor control in the DVL3 model. A,** Schema of the study design for *in vivo* therapy experiment. C57Bl/6 male mice were implanted with (1X10^^6^) DVL3 in the right flank. Once the tumors were established (5 weeks post cells inoculation), mice were randomized and αPD-L1 antibody was dosed at 10mg/kg, 3 times a week, for 2 weeks, as per schema. **B,** Mean tumor volume showing that blockade of the PD-L1 axis does not induce tumor control in the DVL3 tumor model. Data represents tumor growth from groups with at least n=5-8 mice in each. **C,** Individual tumor growth following initiation of treatment on day 0 in the DVL3 tumor model.

**Figure-S3: Fractionated RT** **increases CD40 expression in the TME of DVL3 tumors**. **A,** Representative IHC images for CD40 chromogenic staining in the spleen (positive control) and **B,** in control tumors and tumors treated with RT**. C,** Quantification of intra-tumoral CD40 within the TME following administration of fractionated RT (3x8Gy fractions) at day 1, 7 and 15 post-treatment, demonstrating a significant increase in CD40 expression in the RT-treated group compared to the controls after 15 days. * Denotes p<0.05 as measured using unpaired T-test with Welch’s correction.

**Figure-S4: Nano-string® gene expression analysis in the DVL3 tumors following RT and αCD40 therapy to re-program the TME**. **A-C,** Venn diagrams of DEGs (p < 0.05 Nanostring® data) for tumor treatments versus non-treated tumors. **D,** Gene ontology (GO) analysis of DEGs showing enriched biological processes across the treatment groups compared to the non-treated tumors (clusterProfiler_3.16.1). The number of DEGs is indicated within the brackets.

**Figure S5:**  **Effect of RT and αCD40 combination therapy on myeloid cells population in the DVL3 tumors.** **A,** Modular heat map of myeloid gene signature showing increase in expression of genes associated with myeloid cells in the combination treated tumors. **B,** Representative multiplex IHC images for CD11b+ and Ly6G+ in the DVL3 tumor sections. Mice were treated as per the schema in figure-2, and tumor samples were excised on day 15. Sections were immune stained for pan myeloid marker (CD11b, green) and granulocytic (Ly6G, magenta) positive cells **C,** All three treatment groups resulted in an increase in proportion of CD11b+ cells in the DVL3 tumors compared to non-treated tumors**.** Quantification of intra-tumoral CD11b+ myeloid cells within the TME in the DVL3 tumors showing an increase in overall proportions compared to control. **D,** Quantification of intra-tumoral CD11b+ Ly6G+ myeloid cells demonstrating a significant increase in the proportion of cells in CD40 treated tumors compared to non-treated animal. Data represents the mean + SEM of n=5-8 animals per treatment group. ns, non-significant, ** p<0.005, using ANOVA test and multiple comparison correction applied.

**Figure S6: Systemic effect of administrating RT and α-CD40 therapy in the DVL3 tumor bearing mice.** **A,** Flow gating strategy for profiling T-cells in the peripheral blood from the tumor bearing mice**.** **B,** Expression of CD40L on CD4+ T-cells in the blood of tumor bearing mice showing an increase in MFI. **C,** Expression of CD40L on CD8+ T-cells in the blood showing no significant difference. **D,** Quantification of CD4+ T cells per ml of blood obtained from tumor bearing mice showing a significant increase in cell per ml of blood when treated with RT/CD40 combination therapy. **E,** Quantification of CD8+ T-cells per ml of blood showing a significant increase in combination treatment group. Data represents mean+ SEM of at least 5 mice per treatment group. * Represents statistical significance p<0.05, **P<0.01, ***P<0.001

**Figure S7: Sequential administration of αCD40mAb antibody in combination with RT resulted in enhanced tumor control and survival in both the DVL3 and TRAMP-C1 tumor models.** **A**, Individual tumor growth following initiation of treatment on day 0 in the DVL3 tumor model and **B**, TRAMP-C1 tumor models. Data representative of at least 2 independent experiments with 8-10 mice per treatment group. Mice were administered RT on day 0 and the CD40mAb was administered sequentially from day 7 onwards and subsequently on days 10, 14 and 17.

**Figure S8:** **The effect of concomitant administration of αCD40mAb with fractionated RT in the TRAMP-C1 tumor model. A,** Schema of the study design for *in vivo* therapy experiment. **B**, Kaplan-Meir survival in the TRAMP-C1 tumor model demonstrating improved survival in the combination treated group. Data represents tumor growth from 2 independent experiment with at least n=8-10 mice per treatment group. **, p<0.01, Log-rank (Mantel-Cox) test. **C,** Representative IHC staining for CD4+ and CD8+ T-cells in the TRAMP-C1 model when the anti-CD40mAb was administered concomitantly with fractionated RT. **D,** The proportion of CD8+T-cells in the RT/CD40 treatment was comparable to the group which received αCD40 treatment alone., whereas no significant increase in CD4+ cell was noted compared to non-treated animals. * Denotes p<0.05; **, p<0.005, using ANNOVA and multiple comparison test applied.

**Figure S9:** **Therapeutic efficacy of low-dose fractionated therapy in combination with** **αCD40mAb on in the TRAMP-C1 and DVL3 tumor model.** **A,** Schema of the experimental protocol. **B,** Kaplan-Meir survival data from the experiments conducted in the TRAMP-C1 model showing that the combination of low-dose fractionated RT with αCD40mAb resulted in no significant growth delay or radio sensitisation compared to RT group alone. Data represents survival end points from at least 8 mice per experimental group. **C,** Mean Tumour volume (MTV) data from the experiments conducted in the DVL3 tumour model showing no added benefit of adding αCD40mAb to low dose fractionated RT (5X2Gy) in the DVL3 tumour model. Data presented is mean+ SEM of n=3-4 mice per experimental group.

**Figure-S10**: **Addition of αPD-L1mAb does not improve therapeutic efficacy of RT and αCD40mAb combination therapy in the DVL3 model. A,** Representative IHC staining for PD-L1 showing no significant increase in PD-L1 staining (green) on CK8+ tumor cells (red). **B,** Representative multiplex IHC images for CD4+ and Foxp3+ cells in the excised tumor sections. Mice were treated with RT or αCD40 antibody as monotherapy or in combination and the tumor samples excised on day-15 and sections evaluated for Foxp3 (magenta) and CD4 (green) staining using multiplex immunohistochemistry. **C,** Schema of experiment for evaluating addition of αPD-L1 therapy to RT and αCD40mAb combination treatment. Mice were dosed with blocking antibodies on the days indicated in the schema **D,** Kaplan Meir survival showing no significant difference following addition of αPD-L1 to RT and αCD40 therapy. Kaplan Meir data represents n= 4-7 animals per treatment group.

**Figure-S11: Administration of αCTLA4mAb improves the therapeutic efficacy of RT and αCD40 combination treatment leading to enhanced tumor control in the murine bladder (MB49) tumors.** **A,** Individual tumor growth showing tumor rejection and control in the mice treated with the triple combination therapy**.** C57BL/6 mice were injected with (1X10^6^) MB49 cells in the supraspinal position. Once the tumors were established (4-6 weeks post cell inoculation), mice were randomized and administered RT delivered as 3 daily fractions of 8Gy on days 0,1 &2 respectively. αCD40mAb was administered sequentially from day 7 and subsequently on days 10, 14 & 17. αCTLA-4 therapy was administered as per the schema on days as indicated in **figure 6A**. Data represents growth from at least 7 mice per treatment group. **B,** Re-challenge experimental data demonstrating long-term surviving (LTS) mice rejecting tumor uptake following reinoculation of the DVL3 tumors cells on the contralateral flank of mice. Additional control male mice were implanted at the same time to confirm tumor growth until the control reached the study end point or tumors were close to limit. The data presented as days following inoculation of tumor cells in both the control and LTS mice. Data represents n= 6 control naïve animals and 8 mice from the triple combination group. **, p<0.01, Log-rank (Mantel-Cox) test.
