# Supplementary material for: Reprogramming the immunosuppressive tumor microenvironment results in successful clearance of tumors resistant to radiation therapy and anti-PD-1/PD-L1
Source: Oncoimmunology. 2023 Jun 15;12(1):2223094. doi: 10.1080/2162402X.2023.2223094 (PMC10274532; doi:10.1080/2162402X.2023.2223094)
Supplement: Supplemental Material [file KONI_A_2223094_SM4288.zip › Supplementary Figures REVISION 180523.pptx]

## Slide 1
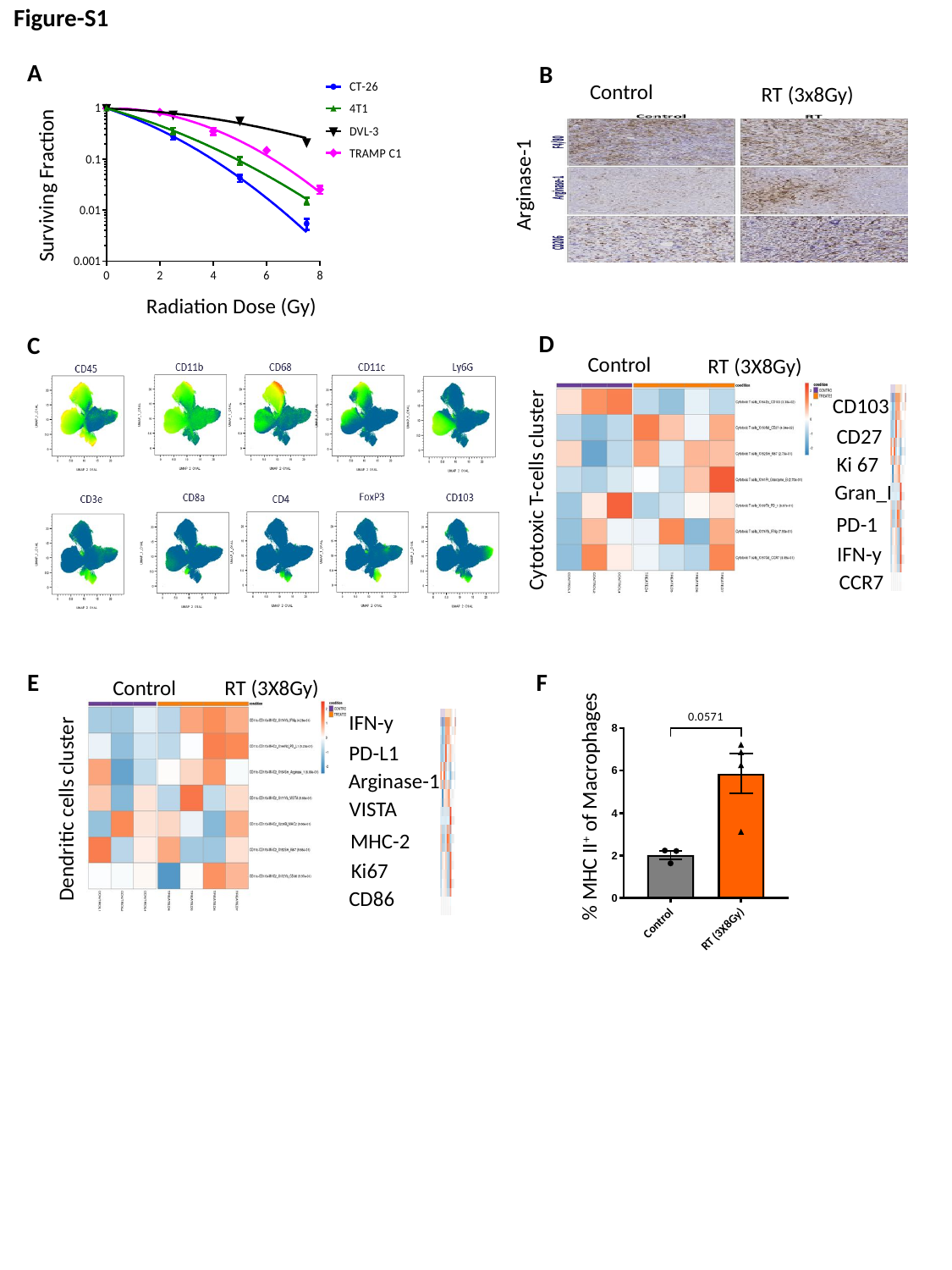

Figure-S1
A
D
C
Control
CD103
CD27
Ki 67
Gran_B
PD-1
IFN-y
CCR7
Cytotoxic T-cells cluster
RT (3X8Gy)
IFN-y
PD-L1
Arginase-1
VISTA
MHC-2
Ki67
CD86
Dendritic cells cluster
 Control RT (3X8Gy)
B
Control
RT (3x8Gy)
Arginase-1
Surviving Fraction
Radiation Dose (Gy)
E
F
% MHC II+ of Macrophages

## Slide 2
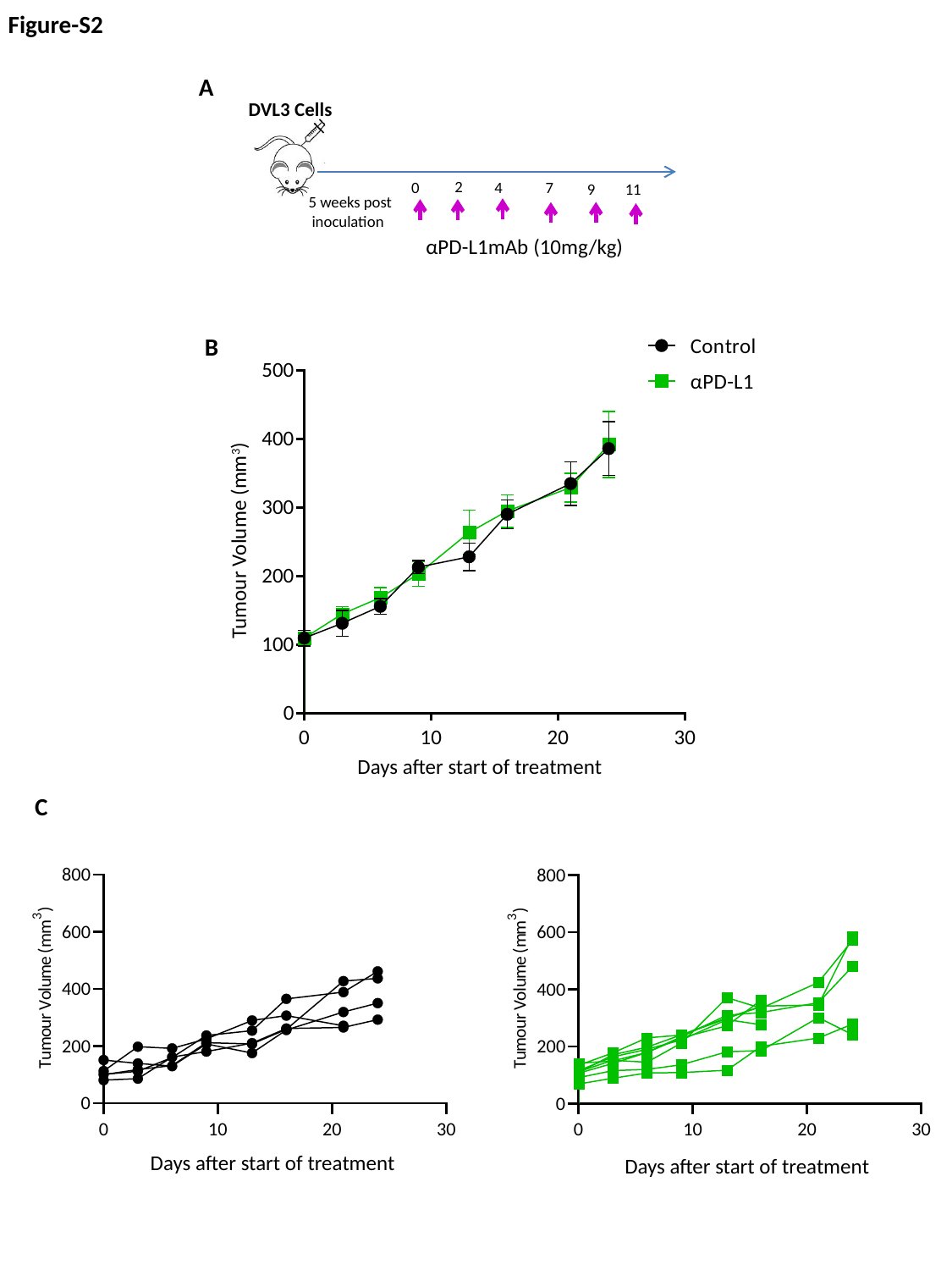

Figure-S2
A
DVL3 Cells
2
4
7
0
9
11
αPD-L1mAb (10mg/kg)
 5 weeks post inoculation
Tumour Volume (mm3)
Days after start of treatment
B
C
Days after start of treatment
Days after start of treatment

## Slide 3
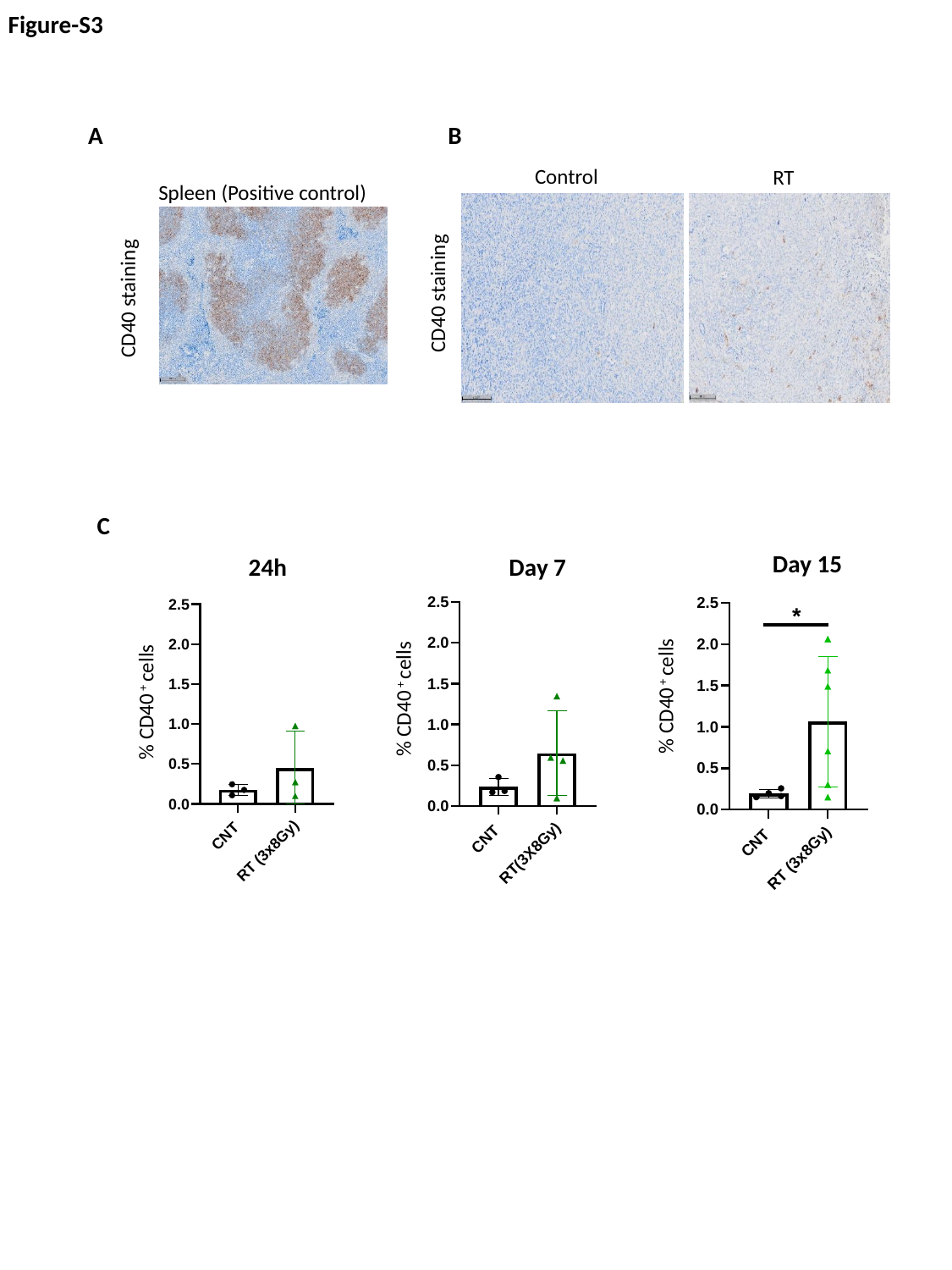

Figure-S3
A
B
Control
RT
Spleen (Positive control)
CD40 staining
CD40 staining
C
Day 15
24h
Day 7
% CD40 + cells
% CD40 + cells
% CD40 + cells

## Slide 4
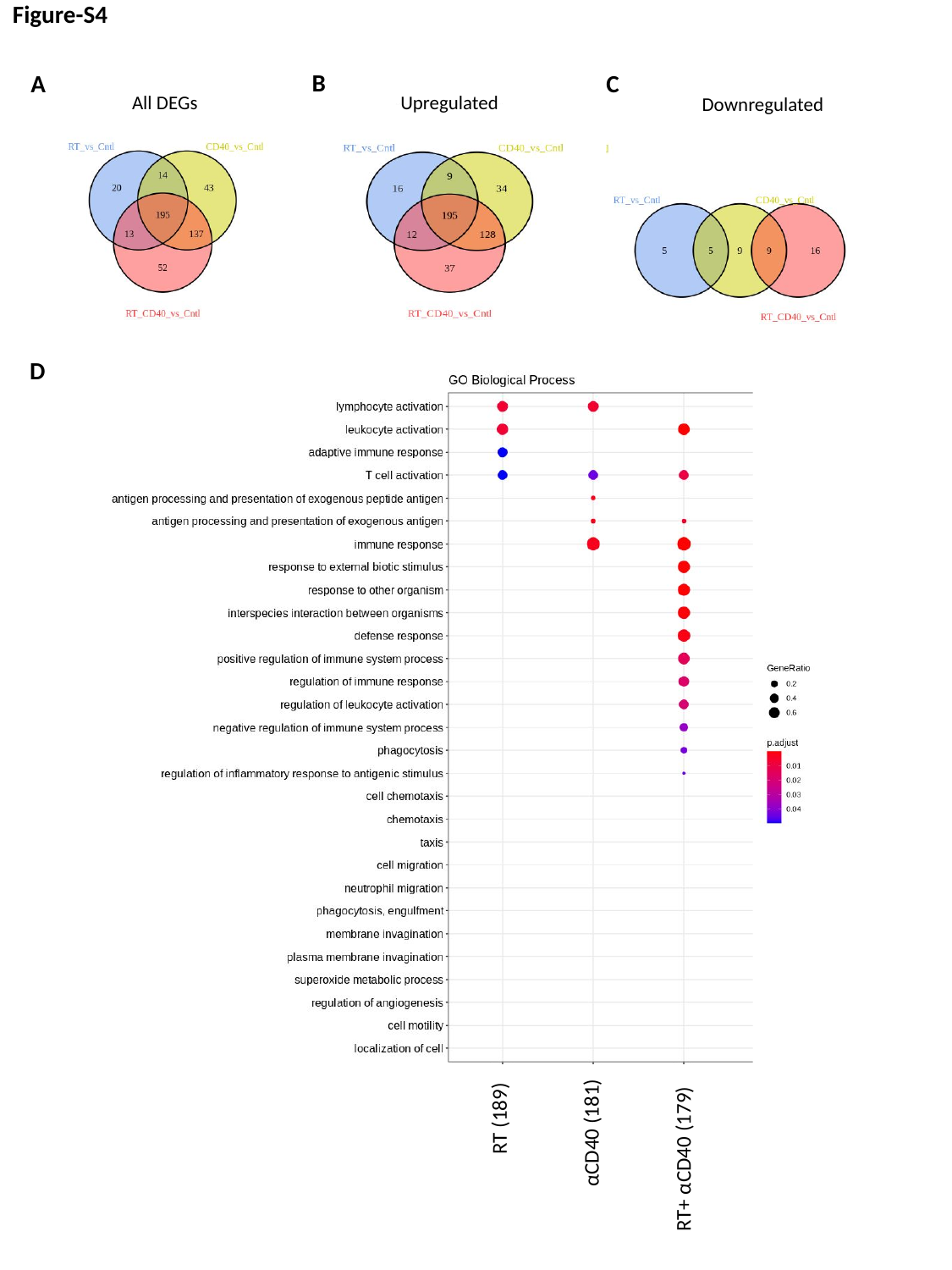

Figure-S4
B
A
C
All DEGs
Upregulated
Downregulated
D
RT (189)
αCD40 (181)
RT+ αCD40 (179)

## Slide 5
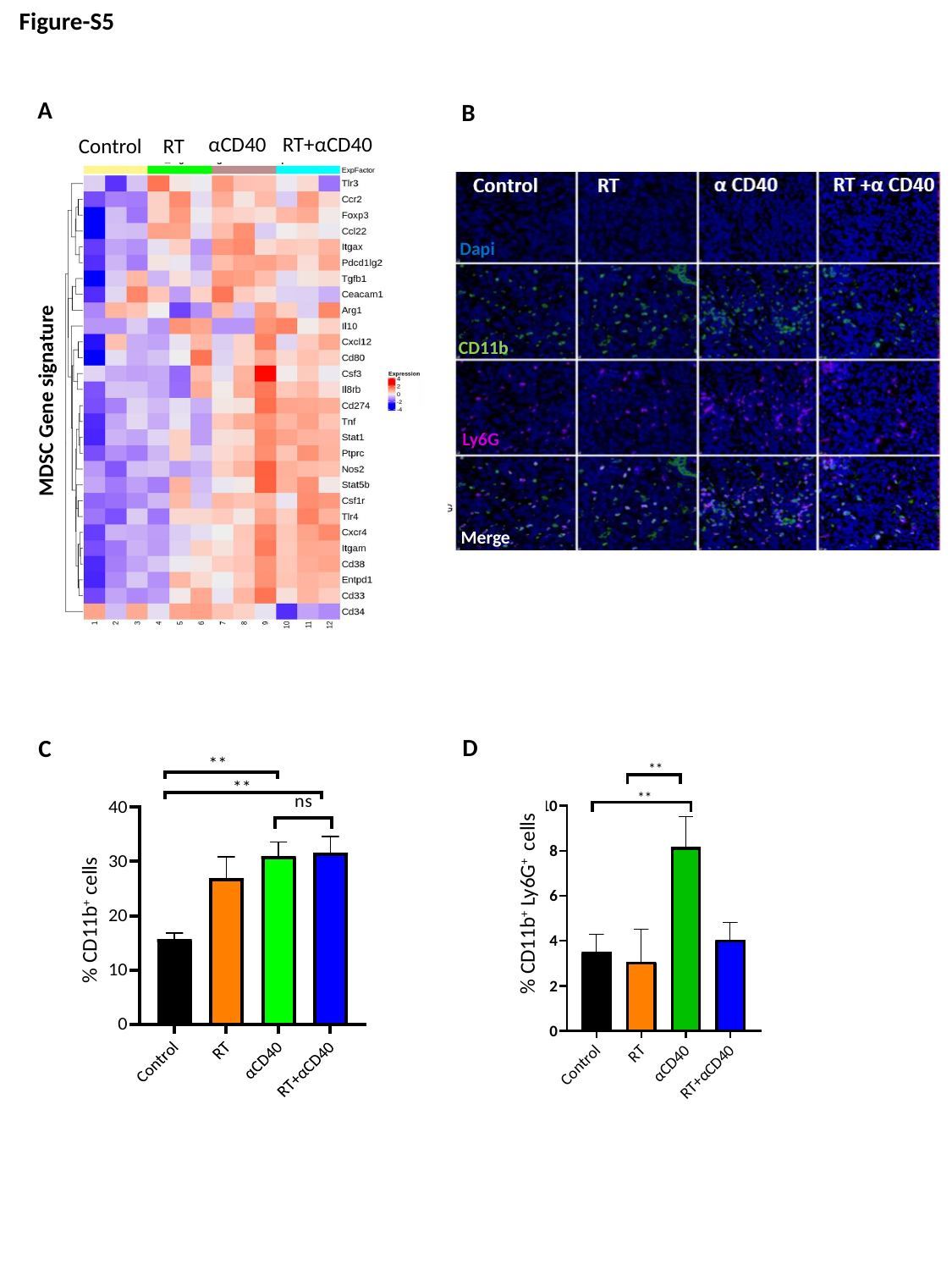

Figure-S5
A
B
αCD40
RT+αCD40
RT
Control
Dapi
CD11b
Ly6G
Merge
MDSC Gene signature
D
C
% CD11b+ Ly6G+ cells
% CD11b+ cells

## Slide 6
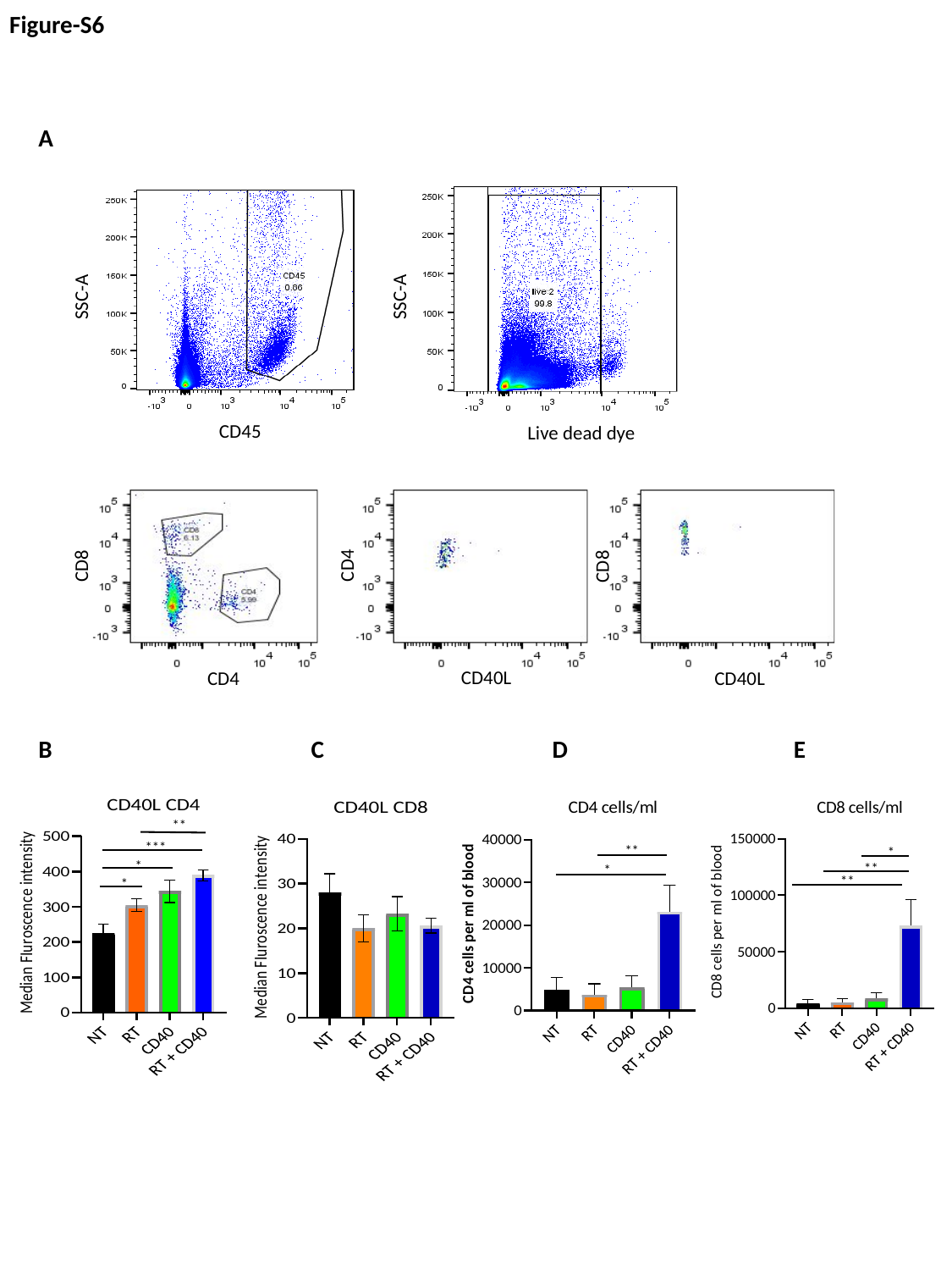

Figure-S6
A
SSC-A
SSC-A
CD45
Live dead dye
CD4
CD8
CD8
CD40L
CD4
CD40L
B
C
D
E
**
***
**
*
*
**
*
**
*

## Slide 7
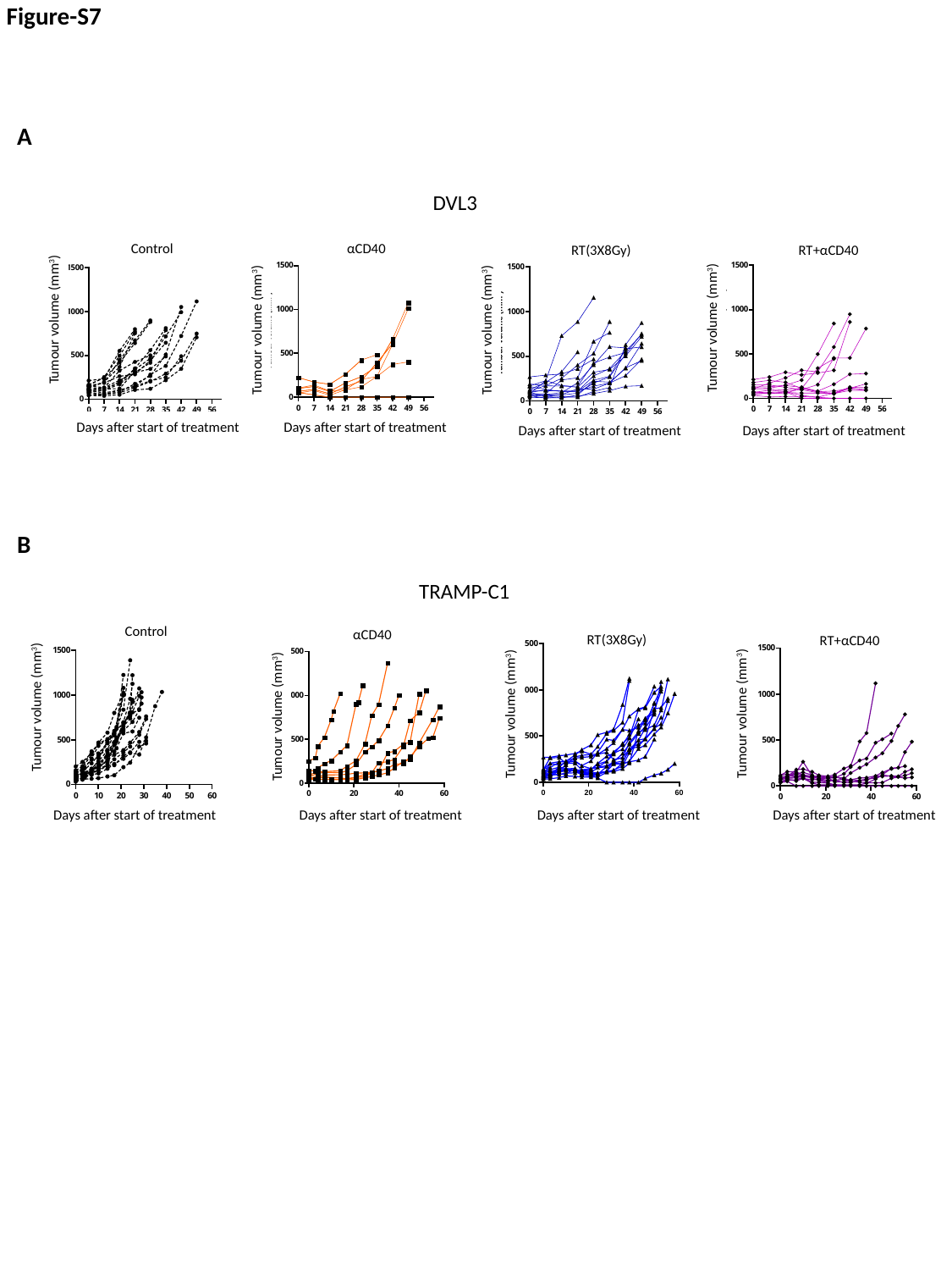

Figure-S7
A
DVL3
Control
αCD40
RT(3X8Gy)
RT+αCD40
Tumour volume (mm3)
Tumour volume (mm3)
Tumour volume (mm3)
Tumour volume (mm3)
Days after start of treatment
Days after start of treatment
Days after start of treatment
Days after start of treatment
B
TRAMP-C1
Control
αCD40
RT(3X8Gy)
RT+αCD40
Tumour volume (mm3)
Tumour volume (mm3)
Tumour volume (mm3)
Tumour volume (mm3)
Days after start of treatment
Days after start of treatment
Days after start of treatment
Days after start of treatment

## Slide 8
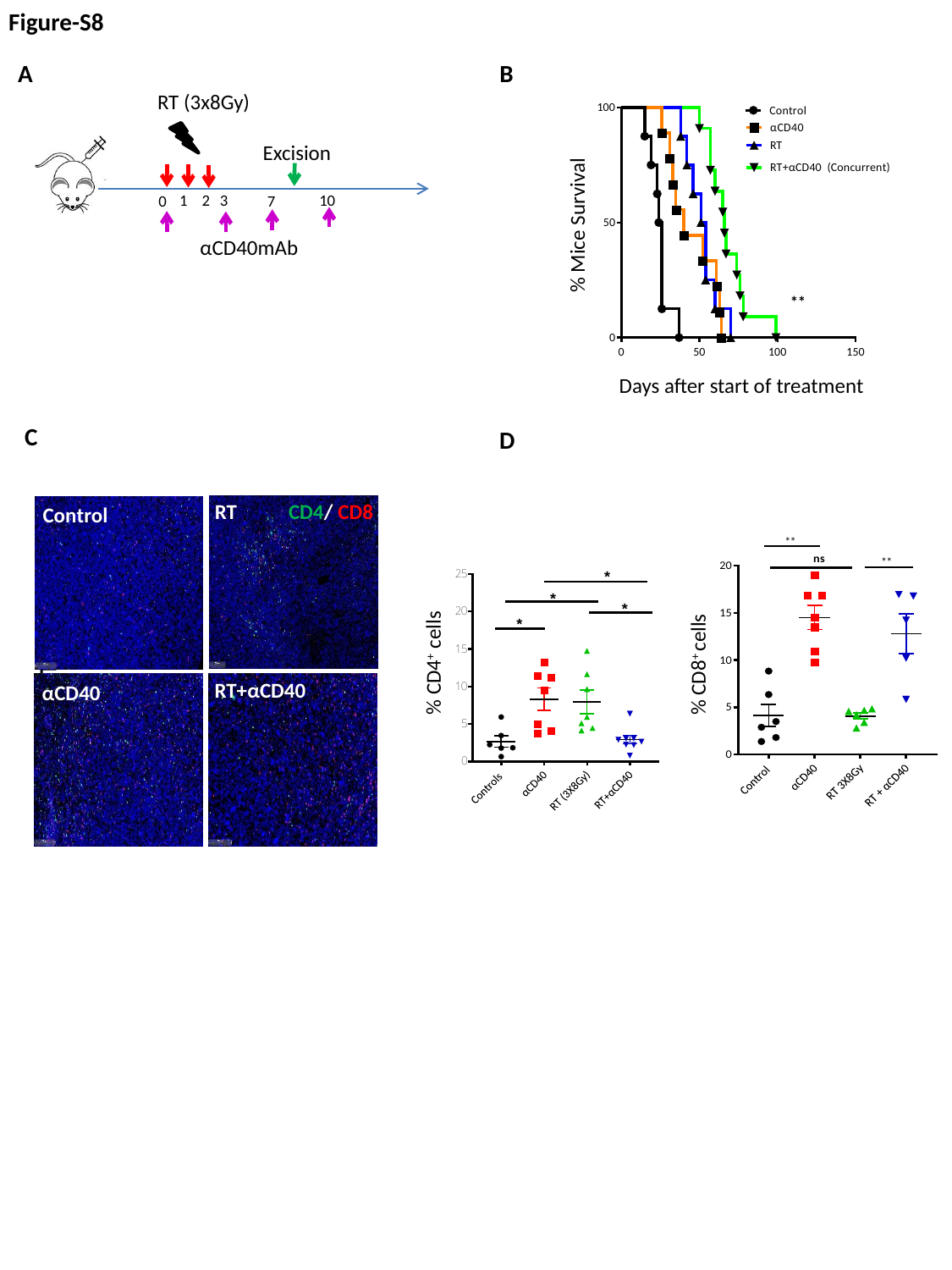

Figure-S8
RT
Control
CD4/ CD8
 Anti-CD40
C
D
TRAMP-C1
A
B
RT (3x8Gy)
10
2
7
0
Excision
3
1
αCD40mAb
% Mice Survival
Days after start of treatment
RT
Control
% CD4+ cells
% CD8+ cells
 Anti-CD40
RT+ Anti-CD40
RT+αCD40
RT+ Anti-CD40
RT+ Anti-CD40
 Anti-CD40
 αCD40

## Slide 9
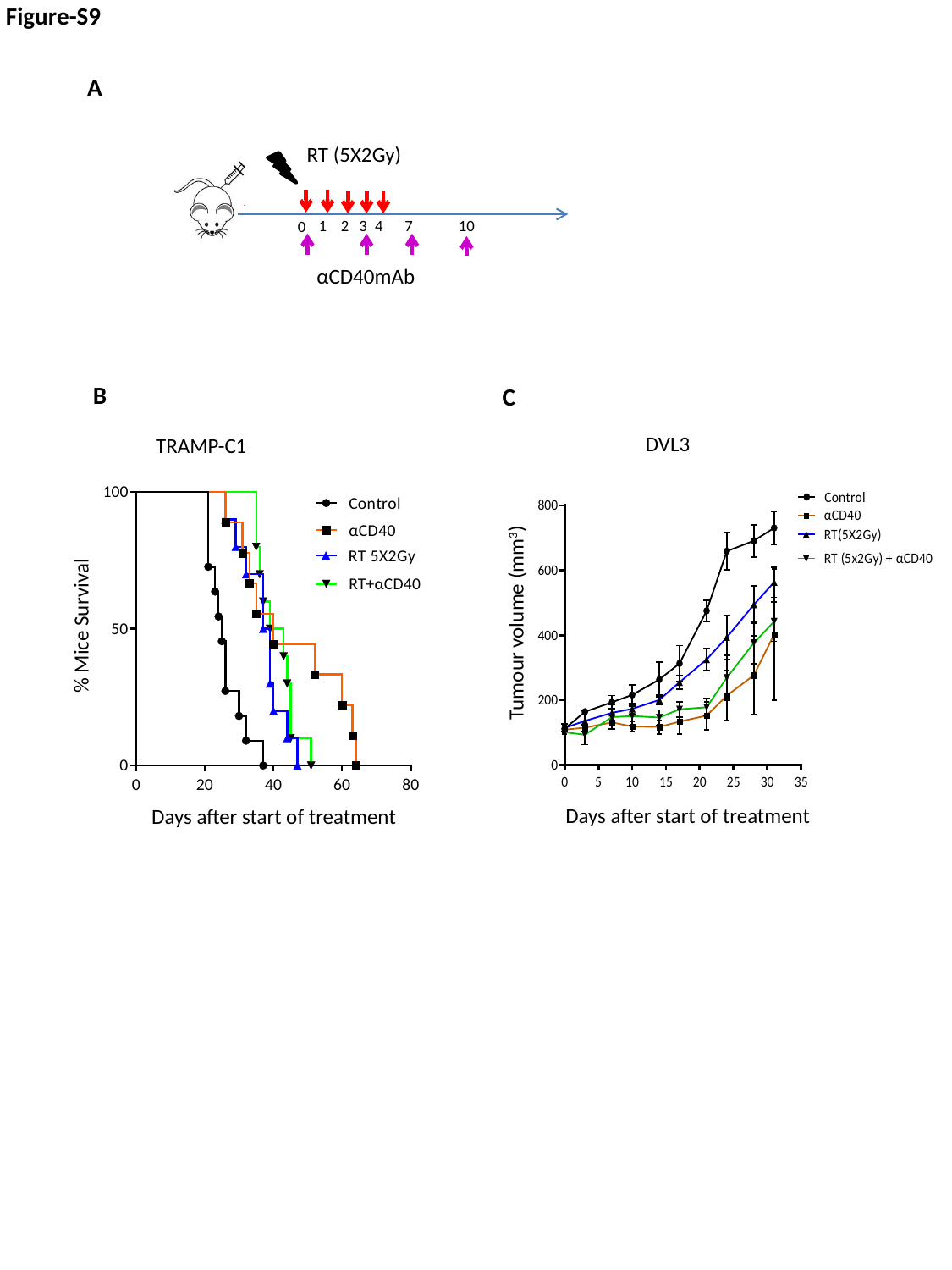

Figure-S9
A
7
10
2
0
RT (5X2Gy)
3
1
αCD40mAb
4
B
C
DVL3
TRAMP-C1
Tumour volume (mm3)
% Mice Survival
Days after start of treatment
Days after start of treatment

## Slide 10
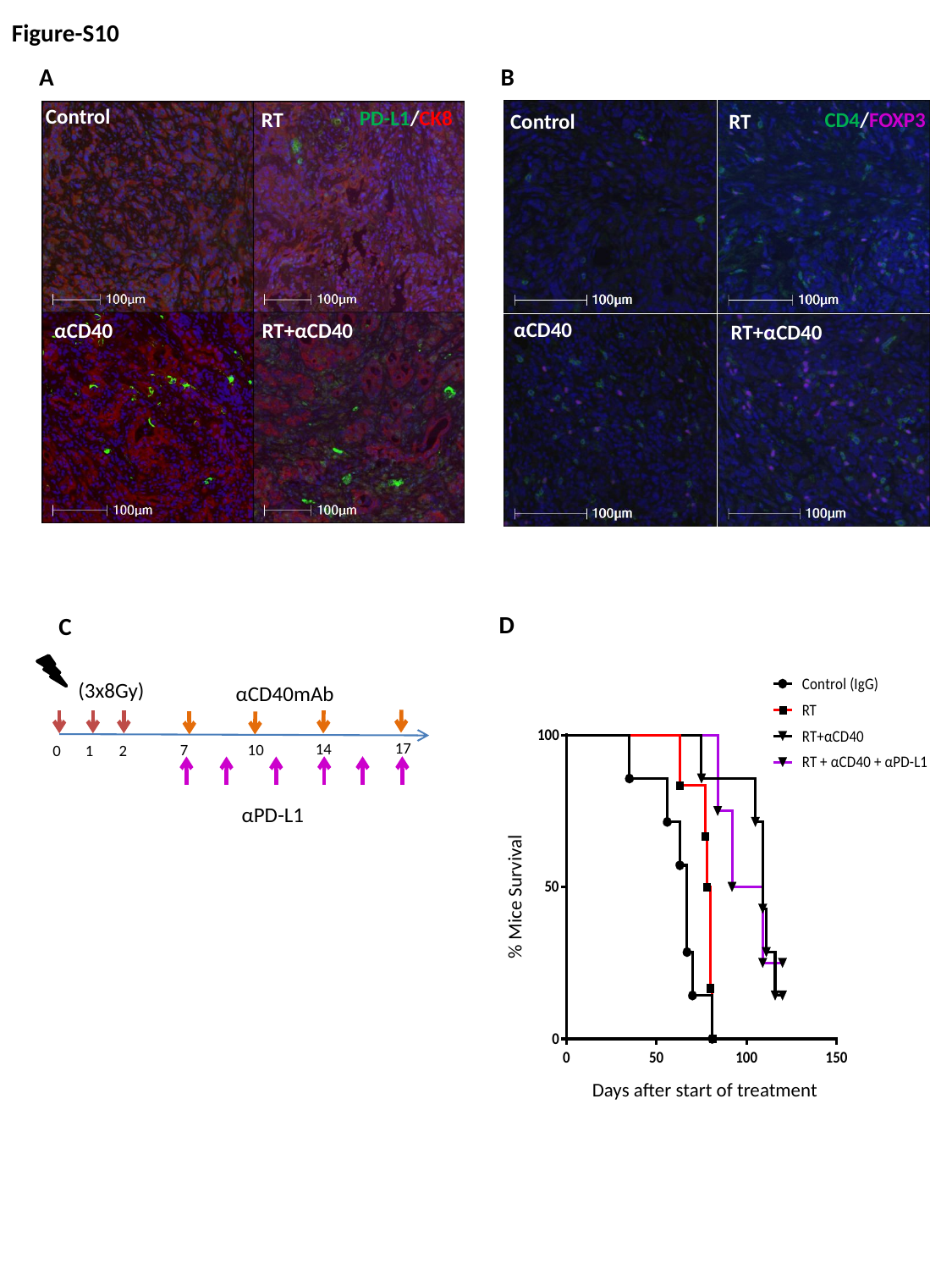

Figure-S10
A
B
Control
PD-L1/CK8
RT
CD4/FOXP3
Control
RT
αCD40
RT+αCD40
αCD40
RT+αCD40
D
C
7
10
0
2
(3x8Gy)
αCD40mAb
17
14
1
αPD-L1
% Mice Survival
Days after start of treatment

## Slide 11
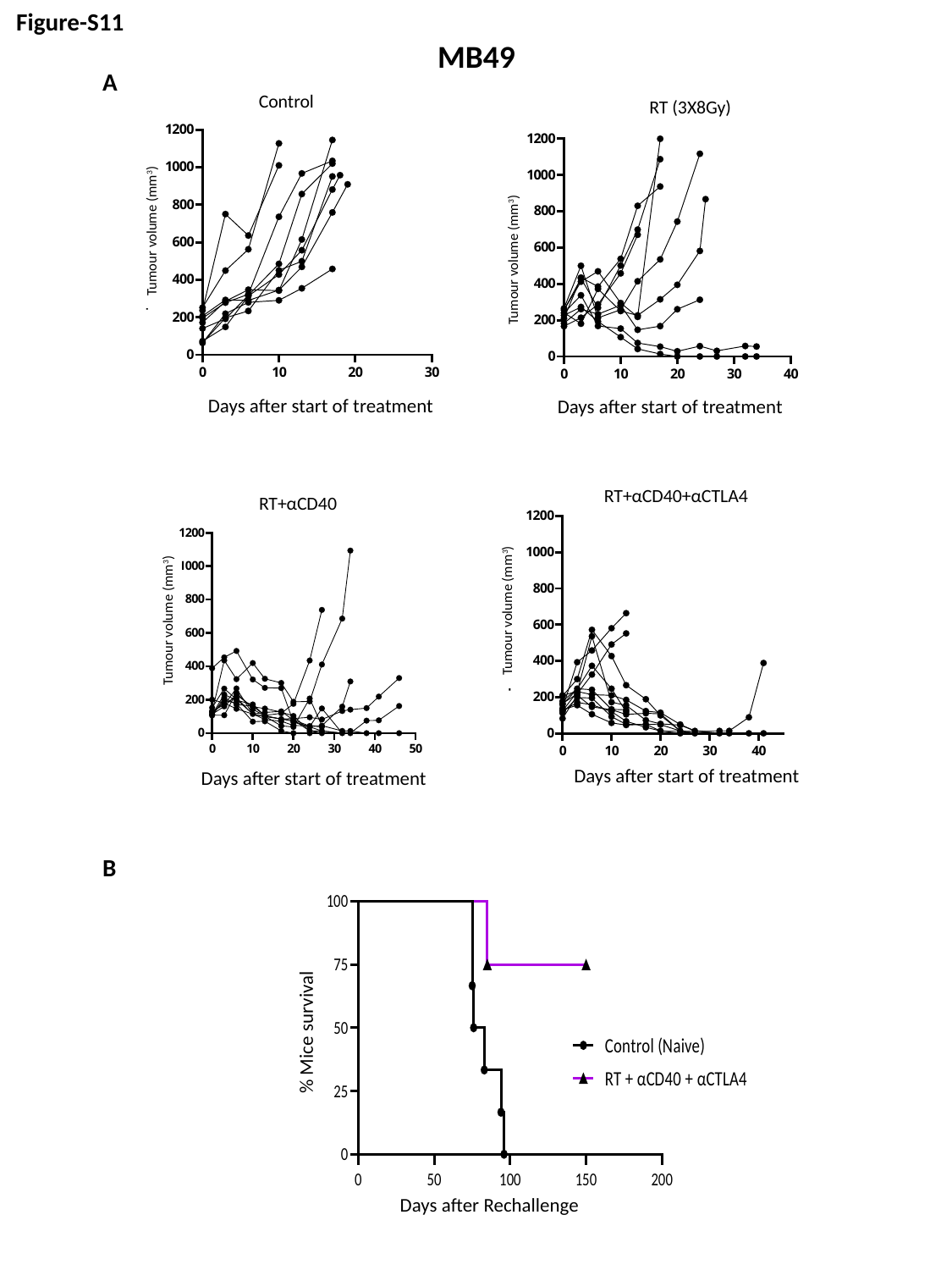

Figure-S11
MB49
A
Control
RT (3X8Gy)
Tumour volume (mm3)
Tumour volume (mm3)
Days after start of treatment
Days after start of treatment
RT+αCD40+αCTLA4
RT+αCD40
Tumour volume (mm3)
Tumour volume (mm3)
Days after start of treatment
Days after start of treatment
B
% Mice survival
Days after Rechallenge

## Slide 12
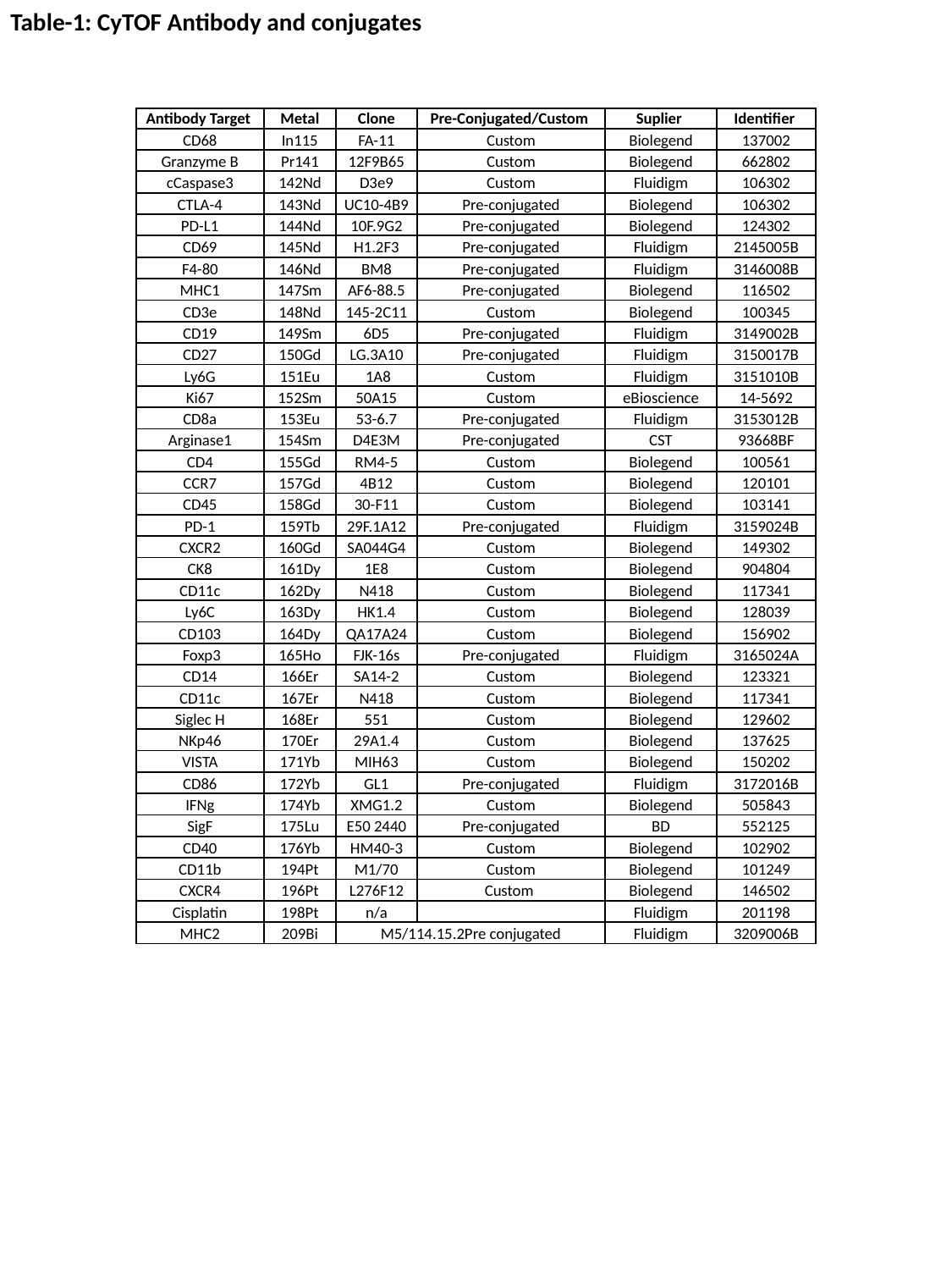

Table-1: CyTOF Antibody and conjugates
| Antibody Target | Metal | Clone | Pre-Conjugated/Custom | Suplier | Identifier |
| --- | --- | --- | --- | --- | --- |
| CD68 | In115 | FA-11 | Custom | Biolegend | 137002 |
| Granzyme B | Pr141 | 12F9B65 | Custom | Biolegend | 662802 |
| cCaspase3 | 142Nd | D3e9 | Custom | Fluidigm | 106302 |
| CTLA-4 | 143Nd | UC10-4B9 | Pre-conjugated | Biolegend | 106302 |
| PD-L1 | 144Nd | 10F.9G2 | Pre-conjugated | Biolegend | 124302 |
| CD69 | 145Nd | H1.2F3 | Pre-conjugated | Fluidigm | 2145005B |
| F4-80 | 146Nd | BM8 | Pre-conjugated | Fluidigm | 3146008B |
| MHC1 | 147Sm | AF6-88.5 | Pre-conjugated | Biolegend | 116502 |
| CD3e | 148Nd | 145-2C11 | Custom | Biolegend | 100345 |
| CD19 | 149Sm | 6D5 | Pre-conjugated | Fluidigm | 3149002B |
| CD27 | 150Gd | LG.3A10 | Pre-conjugated | Fluidigm | 3150017B |
| Ly6G | 151Eu | 1A8 | Custom | Fluidigm | 3151010B |
| Ki67 | 152Sm | 50A15 | Custom | eBioscience | 14-5692 |
| CD8a | 153Eu | 53-6.7 | Pre-conjugated | Fluidigm | 3153012B |
| Arginase1 | 154Sm | D4E3M | Pre-conjugated | CST | 93668BF |
| CD4 | 155Gd | RM4-5 | Custom | Biolegend | 100561 |
| CCR7 | 157Gd | 4B12 | Custom | Biolegend | 120101 |
| CD45 | 158Gd | 30-F11 | Custom | Biolegend | 103141 |
| PD-1 | 159Tb | 29F.1A12 | Pre-conjugated | Fluidigm | 3159024B |
| CXCR2 | 160Gd | SA044G4 | Custom | Biolegend | 149302 |
| CK8 | 161Dy | 1E8 | Custom | Biolegend | 904804 |
| CD11c | 162Dy | N418 | Custom | Biolegend | 117341 |
| Ly6C | 163Dy | HK1.4 | Custom | Biolegend | 128039 |
| CD103 | 164Dy | QA17A24 | Custom | Biolegend | 156902 |
| Foxp3 | 165Ho | FJK-16s | Pre-conjugated | Fluidigm | 3165024A |
| CD14 | 166Er | SA14-2 | Custom | Biolegend | 123321 |
| CD11c | 167Er | N418 | Custom | Biolegend | 117341 |
| Siglec H | 168Er | 551 | Custom | Biolegend | 129602 |
| NKp46 | 170Er | 29A1.4 | Custom | Biolegend | 137625 |
| VISTA | 171Yb | MIH63 | Custom | Biolegend | 150202 |
| CD86 | 172Yb | GL1 | Pre-conjugated | Fluidigm | 3172016B |
| IFNg | 174Yb | XMG1.2 | Custom | Biolegend | 505843 |
| SigF | 175Lu | E50 2440 | Pre-conjugated | BD | 552125 |
| CD40 | 176Yb | HM40-3 | Custom | Biolegend | 102902 |
| CD11b | 194Pt | M1/70 | Custom | Biolegend | 101249 |
| CXCR4 | 196Pt | L276F12 | Custom | Biolegend | 146502 |
| Cisplatin | 198Pt | n/a | | Fluidigm | 201198 |
| MHC2 | 209Bi | M5/114.15.2Pre conjugated | | Fluidigm | 3209006B |

## Slide 13
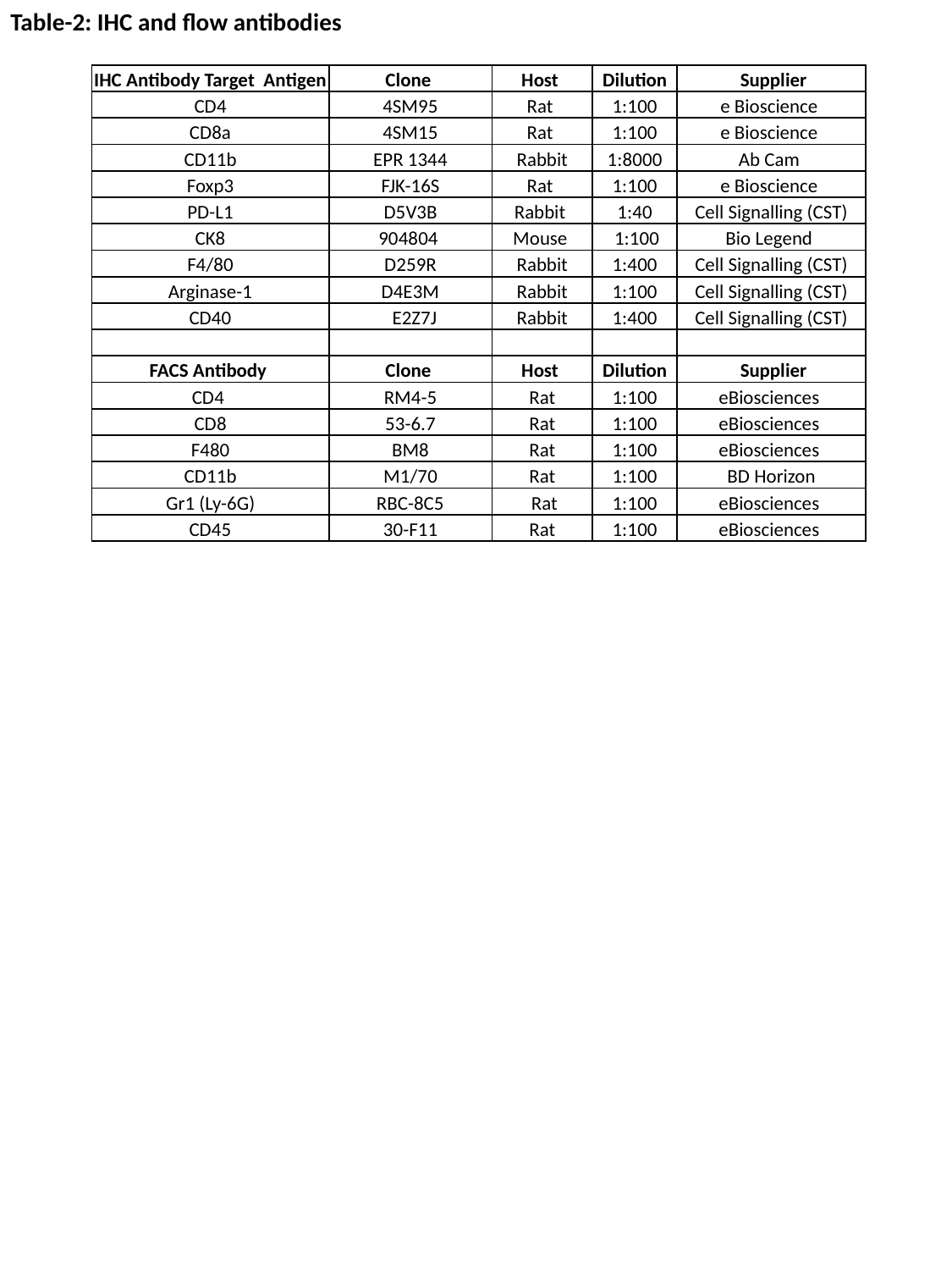

Table-2: IHC and flow antibodies
| IHC Antibody Target Antigen | Clone | Host | Dilution | Supplier |
| --- | --- | --- | --- | --- |
| CD4 | 4SM95 | Rat | 1:100 | e Bioscience |
| CD8a | 4SM15 | Rat | 1:100 | e Bioscience |
| CD11b | EPR 1344 | Rabbit | 1:8000 | Ab Cam |
| Foxp3 | FJK-16S | Rat | 1:100 | e Bioscience |
| PD-L1 | D5V3B | Rabbit | 1:40 | Cell Signalling (CST) |
| CK8 | 904804 | Mouse | 1:100 | Bio Legend |
| F4/80 | D259R | Rabbit | 1:400 | Cell Signalling (CST) |
| Arginase-1 | D4E3M | Rabbit | 1:100 | Cell Signalling (CST) |
| CD40 | E2Z7J | Rabbit | 1:400 | Cell Signalling (CST) |
| | | | | |
| FACS Antibody | Clone | Host | Dilution | Supplier |
| CD4 | RM4-5 | Rat | 1:100 | eBiosciences |
| CD8 | 53-6.7 | Rat | 1:100 | eBiosciences |
| F480 | BM8 | Rat | 1:100 | eBiosciences |
| CD11b | M1/70 | Rat | 1:100 | BD Horizon |
| Gr1 (Ly-6G) | RBC-8C5 | Rat | 1:100 | eBiosciences |
| CD45 | 30-F11 | Rat | 1:100 | eBiosciences |
