# Supplementary material for: Reprogramming the immunosuppressive tumor microenvironment results in successful clearance of tumors resistant to radiation therapy and anti-PD-1/PD-L1
Source: Oncoimmunology. 2023 Jun 15;12(1):2223094. doi: 10.1080/2162402X.2023.2223094 (PMC10274532; doi:10.1080/2162402X.2023.2223094)
Supplement: Supplemental Material [file KONI_A_2223094_SM4288.zip › Supplementary Materials and Methods REVISION 180523.docx]

**Supplementary Materials and Methods**:

***In vitro* Clonogenic Assay**

Cells (25-1600) were seeded in 6 well plates and incubated in complete medium at 37 °C for 4 hours. Plates were irradiated at increasing doses using a Xtrahl® CIX3 cabinet X-ray irradiator at 300Kv at a dose rate of 2.022Gy/minute. The cells were placed at a height of 40 FSD and the radiation was delivered in top-down manner. Cell were then incubated for 8-10 days at 37 °C until they formed visible colonies and fixed with 70% ethanol and stained with 0.4% crystal violet in 70% methanol and counted manually. At least three independent experiments were carried out and the plating efficiency and survival fraction was calculated. Survival fraction in the absence of radiotherapy was corrected to 1. Linear quadratic analysis was performed in GraphPad prism 7 (GraphPad Software, San Diego, CA, USA) software and results were reported as mean ± S.E.M.

**Sample Preparation for Immunohistochemistry and flow cytometry**

Tumor bearing mice were euthanized at indicated time points using standard procedure and in accordance with the current home office legislation, UK. Samples were harvested from mice and tumors were cut into half and either fixed in 4% buffered formalin (Sigma, UK) for 24 hours or collected in RPMI media for tumor disaggregation. The formalin fixed tumor samples were transferred to 70% ethanol the next day and processed to FFPE blocks at the CRUK Manchester Histology Core Facility. To obtain single cell suspensions, tumors were processed using a gentle Macs dissociator and a murine dissociation kit (Miltenyi Biotec, UK). For staining of cells, non-specific binding was blocked with rat anti-CD16/CD32 Fc block on ice for 30 minutes. Cells were incubated with antibodies (**Table-2**) washed in 1% FCS/PBS. For analysis, live cells were gated using vital dye exclusion (Invitrogen) and population phenotyped on FACs Canto (BD Bioscience) and analysed using Flow Jo software. An example of the gating strategy employed is provided in the (**Supplementary Figure S6**).

**Mass cytometry:**

**(A) Antibodies and conjugation**

Antibody information is listed in (**Table-1)**. The antibodies were either purchased pre-conjugated (Fluidigm) or in house conjugated. In-house conjugation was performed using antibody conjugations kits (Fluidgm), with addition of an equal volume of PBS-based antibody stabilisation buffer (Candor Biosciences). To generate cisplatin conjugates, 200μg of antibody was reduced and incubated with 200μl of 400μM monoisotopic cisplatin (BuyIsotope) in C-buffer from the Antibody Conjugation Kits at 37°C for 90 minutes and washed and stored as for the polymer/lanthanide conjugates. Antibodies were titrated in panels by staining samples of known positive and negative controls.

**(B) Extracellular staining**

Tumour samples were dissociated using the gentle mac dissociator using the murine dissociation kit (Miltenyi Biotec) following the manufacturers instruction. The cells were counted and approximately 1 x 10^6^ cells per sample were re-suspended in FACs tube in cell staining medium (Fluidigm). Live cells were spun by centrifuging at 400g for 6 minutes. The disaggregated tumour cell pellet was re- suspended in 100ul IX 198Pt monoisotopic cisplatin (Fluidigm) at a final concentration of 1µM for 1 minutes, followed by vortexing. The suspension was quenched with 2ml of CSM to stop the reaction and centrifuging and subsequent by removal of the supernatant. The cells were incubated in 20µl of 100U/ml heparin sodium salt (Sigma Aldrich) and 1ul of anti-CD16/32 antibody (e-Bioscience) for 5 minutes on ice before adding the remaining master mix of extracellular surface antibodies in 50μl CSM per tube and incubation for 45 minutes. The cells were then washed twice with 3ml of CSM and fixed/permed using FOXP3 Fixation/Permeabilization kit (Thermo Fisher) following manufacturer’s instructions. After permeabilization, the cell pellet was re-suspended in 1ml of 10% v/v DMSO (Sigma Aldrich) in CSM-I (Cell Staining Buffer-Intracellular), consisting of 5 mg/ml BSA and 0.2mg/ml sodium azide in PBS and frozen at -20 until they were ready for barcoding.

**(C) DNA staining and acquisition**

Cells were thawed at room temperature and washed with 2ml cell staining media twice and the pellet re suspended in 3ml of PBS. The pellet for each sample was barcoded using the Cell-ID 20-plex Pd Barcoding Kit (Fluidigm) following manufacturer’s instructions and washing twice with 3ml of CSM buffer. Samples were pooled in 4ml of 1x FOXP3 permeabilization buffer (ThermoFisher) and pelleted. For each sample included in the pooled samples, 20μl of heparin sodium salt in PBS and 1μl of Fc block was added and the sample mixed by gently rocking. After incubating for 5 minutes at RT in the dark, a master mix of intracellular targeting, metal conjugated antibodies (**Table-1**) in CSM were added. After 45 minutes of incubation, the cells were washed twice with 4ml of CSM fixed in 4% paraformaldehyde (PFA) (Thermo Fisher, UK). The sample was vortexed and stored overnight at 4°C.

**(D) DNA staining and acquisition**

On the day of acquisition, 1μl of Cell-ID Iridium Intercalator (Fluidigm) was added for every 3x10^^6^ cells included in the pooled sample for 50 minutes. Following incubation at room temperature, the cells were washed in PBS no more than 4ml at 1000g for 6 minutes. Each cell pellet was washed twice with water and re-suspended at a concentration of 1x106 cells/ml in 15% EQ Four Element Calibration Beads (Fluidigm) in water and thereafter the pellet was filtered twice through 70μm Filcons (BD Biosciences) and acquired on a Helios Mass Cytometer (Fluidigm), using a Super Sampler (Victorian Airship & Scientific Apparatus LLC) at a maximum of 500 events/seconds.

**(E): Data processing and analysis**

FCS files were normalized for signal-drift during the acquisition run using the in-built Helios normalization tool (Fluidigm) and individual sample events deconvoluted using the stand-alone debarcoder, using a Mahalanobis distance of 15 and a minimum barcode separation of 0.26. Individual sample FCS files were uploaded to Cytobank ([www.cytobank.org](http://www.cytobank.org) , Beckman Coulter). As per standard methods, live cell events were selected based on 191Ir positivity and 198Pt negativity. 191Ir+ debris and cell doublets and aggregates were removed based on event length. If possible, target cells were selected by manual biaxial gating: T cell events selected as CD45+CD3+ , further divided in CD8+ cluster for cytotoxic T-cells. Target cells were exported and uploaded to the Cytofkit2 package (Version 2.0.1 or higher). Cells were clustered using Flow SOM and visualised using UMAP projections and expression overlays and exporting cell data with annotated cluster for further downstream analysis. Plotting and statistical analysis was done using R Statistical software and the result files exported from Cytofkit2 using the methods described in (<https://www.bioconductor.org/help/coursematerials/2017/BioC2017/Day2/Workshops/CyTOF/doc/cytofWorkflow_BioC2017workshop.html#differential-cell-population-abundance>). Charts were generated in R studio and source code uploaded on Manchester figshare (<https://figshare.manchester.ac.uk/search>). The data can be accessed using DOI: 10.48420/16826425. The percentage of each immune cell population were calculated from the FlowSOM output. This was done for each sample, and then means and standard deviation were calculated from these percentages according to condition (control and RT treated samples). Differential analysis of marker expression was done using a linear model as described in the above workflow. Heatmaps were plotted using normalised expression, with cluster-marker instances ordered by significance according to adjusted p value. The clusters and associated markers used for each heatmap were as follows: T-cells merging of (Cytotoxic T Cells, T helper cells, and T regs clusters) and looking at the expression of PD-1, CD27, Granzyme B, CD103, Foxp3, IFN-y, Ki67, CCR. We also looked at the Cytotoxic T-cell cluster for expression of PD-1, CD27, Granzyme B, CD103, Foxp3, IFN-y, Ki67, CCR. Dendritic cell cluster for expression of IFN-y, PD-L1, Arginase-1, VISTA, MHC-II, Ki67 and CD86. Comparisons were performed using Kruskal-Wallis test and Dunn’s test for pairwise comparison for the adjusted p-values

**Immunohistochemistry**

For manual staining on the bench, slides were deparaffinised in xylene twice for 5 minutes followed by gradual rehydration in alcohols. Antigen retrieval was performed using pH 6 citrate buffer (Dako, Agilent, UK) in a pressure cooker (Biocare Medica TM), followed by quenching in 3% H202 for 5-minutes. Slides were incubated with 10% serum, prior to incubation with primary antibody (**Table 2**). Primary antibody was detected with either HRP detection kit or biotinylated secondary antibody followed by ABC detection kit (Vector Labs, USA). Slides were briefly incubated in DAB substrate (Vector Labs, USA), washed in water, and counter-stained using haematoxylin. All antibodies, source and concentration used for both multiplex and single plex immunohistochemistry have been listed in **(Table 2)**.

For multiplex staining of mouse FFPE tumour sections, the opal TSA detection system (Akoya Biosciences, USA) was applied to sections following manufacturer’s instruction. Briefly slides were deparaffinised on the Bond Rx followed by epitope retrieval using the Epitope retrieval solution-1 (Leica Biosystem). The endogenous peroxidase was blocked for 5 minutes followed by three washes in wash buffer. The slides were incubated with primary antibodies (**Table 2**) for 60 minutes at 37^0^ degree. Following a set of three wash steps, slides were incubated with an ImmPress® horseradish peroxidase (HRP)-conjugated secondary antibody (Vector Laboratories) for 30 minutes. The slides were subsequently incubated with an opal TSA fluorophore (Akoya Biosciences) for 10 minutes at room temperature. All opal TSA were diluted in IX Amplification reagent (Akoya Biosciences) and used at a final concentration of 1:200. The slides were counterstained with DAPI twice on the Bond Rx platform.

**RNA Extraction for Nanostring and RNA Seq analysis**

Briefly, 2–4 (10uM) sections were transferred into RNAase free microcentrifuge tube. The paraffin was removed by incubating in xylene and ethanol. For lysate and total RNA purification, digestion buffer and proteinase-K was added to the samples as per manufacturer’s instructions (Norgen Kit). The samples were spun briefly followed by transferring the supernatant to a new RNAase free microcentrifuge tube. The RNA containing tubes were incubated for 15 min at 80 °C. The lysates were then passed through RNA purification micro column and centrifuged for 1 min at 14,000 RPM. The micro columns were washed according to manufacturer’s instructions and the RNA eluted using the Elution solution (Norgen Kit).

**Image Acquisition and Analysis**:

Image analysis on digitally scanned slide was performed using the Halo® image analysis software (Indica Labs®). Quantification of the positive cells was determined using either haematoxylin or DAPI staining to identify total cell nuclei. Scoring of positive staining of both chromogen and multiplex slides was performed in a blinded fashion by at least 2 independent researchers. For quantification of co-localisation using Halo ®; High plex FL Version 3.1.and above was used. All image analysis was performed at the CRUK Manchester Institute Imaging computer and data stored at the local network drive.
